# Metabolic Reprogramming by Malat1 Depletion in Prostate Cancer

**DOI:** 10.3390/cancers13010015

**Published:** 2020-12-22

**Authors:** Simona Nanni, Aurora Aiello, Chiara Salis, Agnese Re, Chiara Cencioni, Lorenza Bacci, Francesco Pierconti, Francesco Pinto, Cristian Ripoli, Paola Ostano, Silvia Baroni, Giacomo Lazzarino, Barbara Tavazzi, Dario Pugliese, PierFrancesco Bassi, Claudio Grassi, Simona Panunzi, Giovanna Chiorino, Alfredo Pontecorvi, Carlo Gaetano, Antonella Farsetti

**Affiliations:** 1 Fondazione Policlinico Universitario A. Gemelli IRCCS, Università Cattolica del Sacro Cuore, 00168 Rome, Italy; francesco.pierconti@unicatt.it (F.P.); francesco.pinto@policlinicogemelli.it (F.P.); cristian.ripoli@unicatt.it (C.R.); silvia.baroni@unicatt.it (S.B.); barbara.tavazzi@unicatt.it (B.T.); pierfrancesco.bassi@unicatt.it (P.B.); claudio.grassi@unicatt.it (C.G.); alfredo.pontecorvi@unicatt.it (A.P.); 2 National Research Council (CNR)-IASI, Department of Engineering, ICT and Technologies for Energy and Transportation, 00185 Rome, Italy; aielloaurora@yahoo.it (A.A.); agnese.re@iasi.cnr.it (A.R.); chiara.cencioni@cnr.it (C.C.); simona.panunzi@biomatematica.it (S.P.); 3 Department of Translational Medicine and Surgery, Università Cattolica del Sacro Cuore Rome, 00168 Rome, Italy; chiara.salis@unicatt.it (C.S.); lorenza.bacci2@unibo.it (L.B.); dariopugliese87@gmail.com (D.P.); 4 Fondazione Edo ed Elvo Tempia, 13900 Biella, Italy; paola.ostano@fondazionetempia.org (P.O.); giovanna.chiorino@fondazionetempia.org (G.C.); 5 UniCamillus-Saint Camillus International University of Health Sciences (Università Cattolica del Sacro Cuore Rome), 00131 Rome, Italy; giacomo.lazzarino@unicamillus.org; 6 Istituti Clinici Scientifici Maugeri, 27100 Pavia, Italy

**Keywords:** prostate cancer, metabolism, metabolic reprogramming, transcription, MALAT1, long non-coding RNA, biomarkers, predictive model, precision medicine

## Abstract

**Simple Summary:**

Prostate cancer (PCa) is one of the most common cancers in developed countries, being the second leading cause of cancer death among men. Surgery is the primary therapeutic option, but about one-third of patients develop a recurrence within ten years, for which successful therapy is unavailable. Based on these observations, it has become urgent to develop novel molecular tools for predicting clinical outcome. Here, we focus on one of the best characterized cancer-associated long non-coding transcripts, namely metastasis-associated lung adenocarcinoma transcript 1 (MALAT1). This study highlighted a novel role for MALAT1 as a controller of prostate cancer metabolism. MALAT1 silencing caused a metabolic rewire in both experimental models adopted, prostate cancer cell lines, and organotypic slice cultures derived from surgical specimens. PCa cells upon MALAT1 silencing revert their phenotype towards glycolysis, which is characteristic of normal prostate cells. In this regard, MALAT1 targeting may represent a promising diagnostic tool and a novel therapeutic option.

**Abstract:**

The lncRNA metastasis-associated lung adenocarcinoma transcript 1 (MALAT1) promotes growth and progression in prostate cancer (PCa); however, little is known about its possible impact in PCa metabolism. The aim of this work has been the assessment of the metabolic reprogramming associated with MALAT1 silencing in human PCa cells and in an ex vivo model of organotypic slice cultures (OSCs). Cultured cells and OSCs derived from primary tumors were transfected with MALAT1 specific gapmers. Cell growth and survival, gene profiling, and evaluation of targeted metabolites and metabolic enzymes were assessed. Computational analysis was made considering expression changes occurring in metabolic markers following MALAT1 targeting in cultured OSCs. MALAT1 silencing reduced expression of some metabolic enzymes, including malic enzyme 3, pyruvate dehydrogenase kinases 1 and 3, and choline kinase A. Consequently, PCa metabolism switched toward a glycolytic phenotype characterized by increased lactate production paralleled by growth arrest and cell death. Conversely, the function of mitochondrial succinate dehydrogenase and the expression of oxidative phosphorylation enzymes were markedly reduced. A similar effect was observed in OSCs. Based on this, a predictive algorithm was developed aimed to predict tumor recurrence in a subset of patients. MALAT1 targeting by gapmer delivery restored normal metabolic energy pathway in PCa cells and OSCs.

## 1. Introduction

Prostate cancer (PCa) is one of the most frequently diagnosed cancers in males, and its increasing worldwide development and progression are open areas of research [1,2]. Recent advances in understanding cancer metabolism might offer more effective therapeutic approaches. In the prostate, healthy cells have a relatively inefficient tricarboxylic acid cycle (TCA)-dependent metabolism, relying more on glycolysis and fatty acid oxidation for energy production [3,4]. This condition is physiological and associated with citrate synthesis and secretion. Citrate is an essential component of the seminal liquid, extensively released by prostate epithelial cells, escaping its processing through the TCA cycle. Indeed, citrate, produced by the first reaction of the mitochondrial Krebs’ cycle that is not converted to isocitrate because of the inhibition of mitochondrial enzyme aconitase [5]. The increased mitochondrial citrate concentration is rapidly and actively secreted in the cytoplasm first and in the extracellular milieu next. The mitochondrial and plasma membrane citrate transporter isoforms are expressed in the prostate epithelial cells (_m_SLC25A1 and _pm_SLC25A1) [6]. Due to the TCA cycle’s truncation and the consequently reduced electron transport chain (ETC) and oxidative phosphorylation (OXPHOS), normal prostate epithelial cells mainly rely on the increased glycolytic rate to foster ATP production [4,7].

Hence, prostate normal epithelial cells’ metabolism partly resembles that of cancer cells, which often exhibit elevated glycolysis for energy and biomass production. Some tumors reduce their energy rate production in favor of prolonged resistance to physical damages or chemotherapeutic agents, a phenomenon called the Warburg effect. For this reason, aerobic glycolysis is not considered a metabolic hallmark of PCa [5,8,9].

The advent of next-generation sequencing methods fueled the discovery of multiple non-coding RNA (ncRNA) transcripts with a direct implication in cell biology, metabolism, and homeostasis, cancer growth, and progression, as well as many other pathophysiological conditions [9]. It appears that long (>200 nt) ncRNAs (lncRNAs) can significantly reprogram cancer cell metabolism by regulating master signaling pathways, suggesting that they might be of potential interest for novel therapeutic strategies [10,11]. Recently, intensive research has been devoted to the molecular dissection of lncRNAs as landmarks of cellular programs active in cancer by providing prognostic value or even foreseeable therapeutic options [12]. Although it is well known that altered cellular metabolism is a significant adaptation of cancer cells to accommodate the high demand for macromolecules during rapid proliferation [9,12,13], the involvement of non-coding RNAs in tumor metabolism is far from being elucidated. While many studies link microRNAs and oncogenic transcriptional regulatory networks involved in cancer cells’ metabolic reprogramming [12], lncRNA’s role in this process has emerged only recently. For example, a lncRNA, the five prime to Xist (Ftx), a regulator of the peroxisome proliferator-activated receptor γ (PPARγ), is overexpressed in hepatocarcinoma (HCC), enhancing glucose consumption, cell proliferation, migration, and invasion [14]. The lncRNA neighbor of BRCA1 gene 2 (NBR2) is known to activate adenosine monophosphate-activated protein kinase (AMPK) in human kidney and breast cancer cell lines, where the uptake of glucose increases in association with GLUT1 transcription [15]. Of interest, different lncRNAs such as H19, GAS5, and LINC00092 can modulate glycolytic enzymes in cancer cell [16,17,18].

The metastasis-associated lung adenocarcinoma transcript 1 (MALAT1) is one of the earliest identified lncRNAs, and its role in cancer as a promoter of tumor progression and metastasis is well-defined [19]. In colorectal cancer, MALAT1 has been associated with increased proliferation and migration [20]; and recently has been shown to regulate the metabolic transcription factor TCF7L2, which promotes metabolic reprogramming relevant for HCC tumor progression [21]. In oesophageal cancer, MALAT1 promotes epithelial-to-mesenchymal transition [22], while in lung and ovarian cancer, it might confer resistance to pharmacological treatment [23,24]. Despite a large body of knowledge about MALAT1 contribution to cellular functions, its involvement in cellular metabolism regulation is still poorly characterized. Relevant to PCa, MALAT1 seems involved in lipid metabolism. In the hepatocarcinoma cell line HepG2, MALAT1 expression increases nuclear localization of the transcription factor sterol regulatory element-binding protein 1. This factor upregulates the expression of genes of lipid synthesis pathways, including the stearoyl CoA desaturase 1c, the fatty acid synthase, the acetyl-CoA carboxylase 1, and ATP citrate synthase [25]. MALAT1 has been reported as highly expressed in HepG2 cells, and its silencing inhibited proliferation, migration, and invasion [26]. However, whether this effect implicated metabolic reprogramming remained unexplored.

In the present study, we investigated the effect of MALAT1 knockdown in PCa cells and organotypic slice cultures (OSC) obtained from human primary PCa samples, an in vitro setting to study human PCa biology at the single patient level. All data collected in OSCs have been compared with those obtained from a subset of well-characterized cell lines with an aggressive/metastatic phenotype.

Our findings suggest that MALAT1 might harness glucose metabolism with consequences for pyruvate catabolism, the TCA cycle, and PCa energy production. In our hands, MALAT1 targeting determines changes in PCa metabolism resembling that of normal epithelial cells. This effect reveals a novel property of MALAT1 as a regulator of metabolic enzymes potentially useful to predict the patient’s outcome.

## 2. Results

### 2.1. Metabolic Perturbation upon MALAT1 Depletion in Prostate Cancer Cell Lines

**Figure 1:**
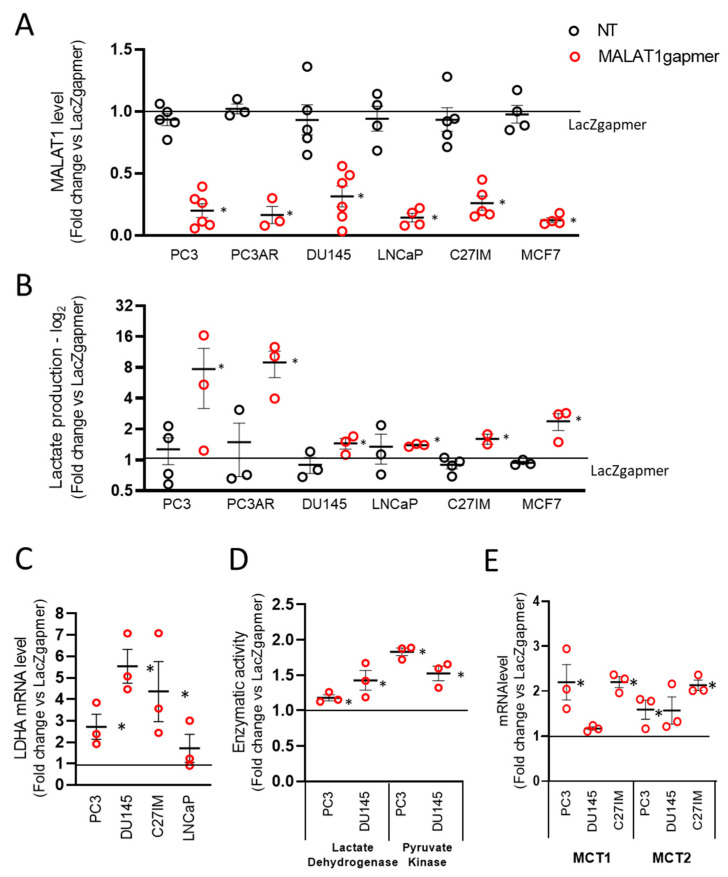
Effects of metastasis-associated lung adenocarcinoma transcript 1 (MALAT1) depletion in prostate cancer cells. (**A**) MALAT1 expression was quantified by qRT-PCR in five different prostate cancer (PC3 *n* = 6, PC3AR *n* = 3, DU145 *n* = 6, LNCaP *n* = 4, and C27IM *n* = 5) and one breast cancer (MCF7 *n* = 4) cell lines at 72 h after transfection with specific (MALAT1), control (LacZ), or no (NT) gapmers. (**B**) Lactate concentration determined by a colorimetric assay in the cell culture supernatant of PC3, PC3AR, DU145, LNCaP, C27IM, and MCF7 cells after MALAT1 gapmerization as in panel A (*n* = 3 or 4). Samples were collected after 48 h from transfection. (**C**,**E**) mRNA levels of lactate dehydrogenase A (LDHA, *n* = 3, C) and monocarboxylate transporter 1 and 2 (MCT1 and MCT2, *n* = 3, D) were quantified by qRT-PCR in PCa cells at 72 h after transfection with MALAT1 or LacZgapmers. (**D**) Enzymatic activity of lactate dehydrogenase and pyruvate kinase measured in PC3 (*n* = 3) cells 24 h after transfection with MALAT1 or LacZgapmers. Results are depicted as fold induction vs. LacZgapmer taken as 1 (black straight line). Individual values with mean +/−SEM are shown (red circles for MALAT1 gapmer, black circles for untreated cells, NT). Non-parametric paired two-tailed Student’s *t*-test determined statistical significance. * *p* ≤ 0.05 MALAT1gapmer vs. LacZgapmer.

To explore the role of MALAT1 in PCa glucose metabolism, we devised a MALAT1 silencing strategy using gapmers [27], which reproducibly depleted the MALAT1 transcript in PCa cell lines (Figure 1A). To verify that the gapmers-dependent targeting was not limited to PCa cells, we also included the breast cancer cell line MCF7.

In this experimental setting, depletion of MALAT1 occurred efficiently, rapidly, and lasted for at least 72 h after transfection as determined by LacZ control gapmers and MALAT1 expression in non-transfected proliferating cells (Figures S1A, S2A and Figure 1A). Transfection of gapmers affected MALAT1 expression with regulatory consequences on the entire MALAT1 genetic locus, as qPCR demonstrated for the nuclear-enriched abundant transcript 1 (NEAT1) and the MALAT1 broadly expressed natural antisense transcript TALAM1. In all PCa cell lines analyzed, NEAT1 and TALAM1 transcripts were upregulated upon MALAT1 depletion, except for TALAM1 in DU145 cells (Figure S1B). Next, we examined the effects of MALAT1 downregulation on cell morphology, proliferation, and death in PC3 cells. Figure S1C,D demonstrates that MALAT1 silencing caused: (i) A change in cell morphology with the appearance of elongated cells and small round-looking ones compared to the parental or LacZgapmerized cells (Figure S1C); (ii) growth arrest, and (iii) cell death increase at 72 h post-transfection (Figure S1D, left and right panel, respectively).

From a metabolic point of view, healthy epithelial prostate cells primarily rely on energy production derived from lipids, fatty acids catabolism, and glycolysis. In this environmental setting, the contribution of oxidative phosphorylation is relatively modest [3,4]. To investigate whether MALAT1 plays a role in the metabolism of PCa-derived cells, we measured the lactate produced in 5 prostate and one breast cancer-derived cell line upon MALAT1 depletion (Figure 1B). Measurement of basal lactate production in the extracellular medium of all cell lines showed that in LNCaP and MCF7 cells, there was a trend toward an increased rate of lactate synthesis as compared to the other cells tested (Figure S2A). We observed a consistent lactate accumulation in the extracellular medium in MALAT1-depleted cells, although this effect was more pronounced in naïve or androgen receptor (AR)-reconstituted PC3 cells. Additional measurements performed in PC3 cells by spectrophotometric analysis confirmed this finding was yielding average values of LacZ 2936 µmol/10^6^ cells ± 0.197 and MALAT1 3809 µmol/106 cells ± 0.291 *p* ≤ 0.05.

In line with these results, we observed an upregulation in lactate dehydrogenase A (LDHA) mRNA paralleled by its enzymatic activity (Figure 1C,D). A similar effect was observed for monocarboxylate transporter 1 and 2 (MCT1 and MCT2) mRNAs (Figure 1E). Figure 1D shows increased lactate dehydrogenase (left) and pyruvate kinase (PK) enzymatic activity in MALAT1-silenced PC3 and DU145. This evidence corroborates the acceleration of the glycolytic rate in MALAT1-silenced cells. In this context, MALAT1 silencing changed the concentration of oxidized (NAD+) and reduced (NADH) nicotinamide adenine dinucleotide. Table S1 shows that deproteinized extracts of PC3 cells depleted of MALAT1 had comparable NAD+ concentration. However, we observed 1.63 times higher NADH values than in naïvePC3 cells (*n* = 4, *t*-test *p* < 0.05). Consequently, significant differences were recorded when calculating the NAD+/NADH ratio (Table S1). This value significantly decreased 1.42 times in MALAT1-silenced PC3 compared to controls.

#### 2.1.2. Gene Profiling after MALAT1 Depletion

**Figure 2:**
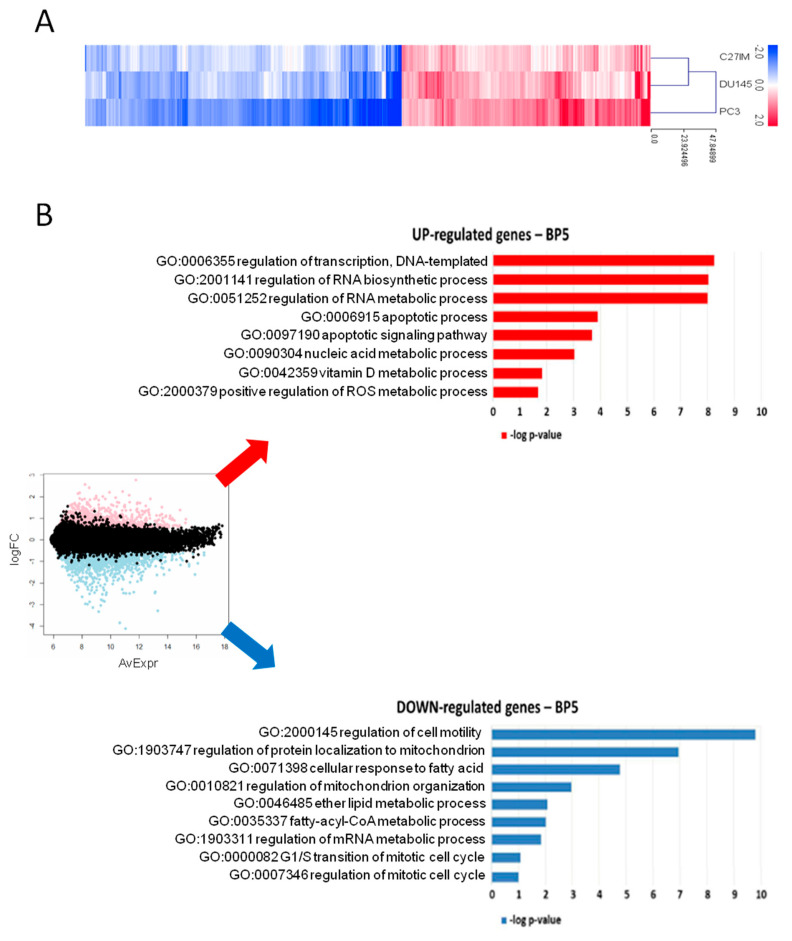
Gene profiling after MALAT1 silencing. (**A**) Heatmap of differentially regulated transcripts (log2 FC > 0.58, namely FC > 1.5; *p* < 0.01) in PC3, DU145, and C27IM cell lines transfected with MALAT1gapmer vs. LacZgapmer (2 biological replicates each, with dye-swap technical replicates). Cell line means log2 FC are represented with red stripes for upregulated genes (*n* = 1237) and blue for downregulated genes (*n* = 1574), as listed in Table S2. (**B**) MA plot with differentially regulated genes highlighted in red and blue (left), and most representative categories of overrepresented biological processes in overexpressed genes (red histograms) and underexpressed genes (blue histograms), according to gene ontology enrichment analysis (right). Histogram length is proportional to the statistical significance of the enrichment (−log10 of enrichment *p*-value).

In light of the evidence that MALAT1 depletion influenced PCa metabolism, we investigated the effect of its reduction on the transcriptome of three different PCa cell lines: C27IM derived from an aggressive-primary tumor, DU145, and PC3 derived from metastatic lesions exhibiting different MALAT1 basal level (Figure S2A). Transfection with MALAT1 gapmer resulted in a highly efficient MALAT1 depletion, ranging from 85% to 95% (Figure S2B). These samples were profiled on the Agilent platform (SurePrint G3 Human Gene Expression v3, 8 × 60K Microarrays (Agilent, Santa Clara, CA, USA), which allowed for the analysis of about 58,000 transcripts, including long non-coding ones. Figure 2A and Table S2 show the genes with expression fold change in MALAT1-depleted vs. controls higher than 1.5 and *p*-value less than 0.01, obtained by linear models for microarray analysis within a bioconductor. Gene ontology (GO) enrichment analysis highlighted that in metastatic cell lines, MALAT1 depletion determined induction of genes involved in biological processes associated with RNA synthesis/metabolism, autophagy, and apoptosis, and compatible with the activation of potential “anticancer pathways” [28] (Figure 2B, upper red panel). Among the downregulated pathways, genes in cancer-associated pathways, transcriptional deregulation, cell cycle, pyruvate metabolism, propanoate metabolism, and PI3K-AKT signaling were overrepresented. This finding depicts a picture compatible with a compromised cell cycle and altered cell metabolism (Figure 2B, lower blue panel).

**Figure 3:**
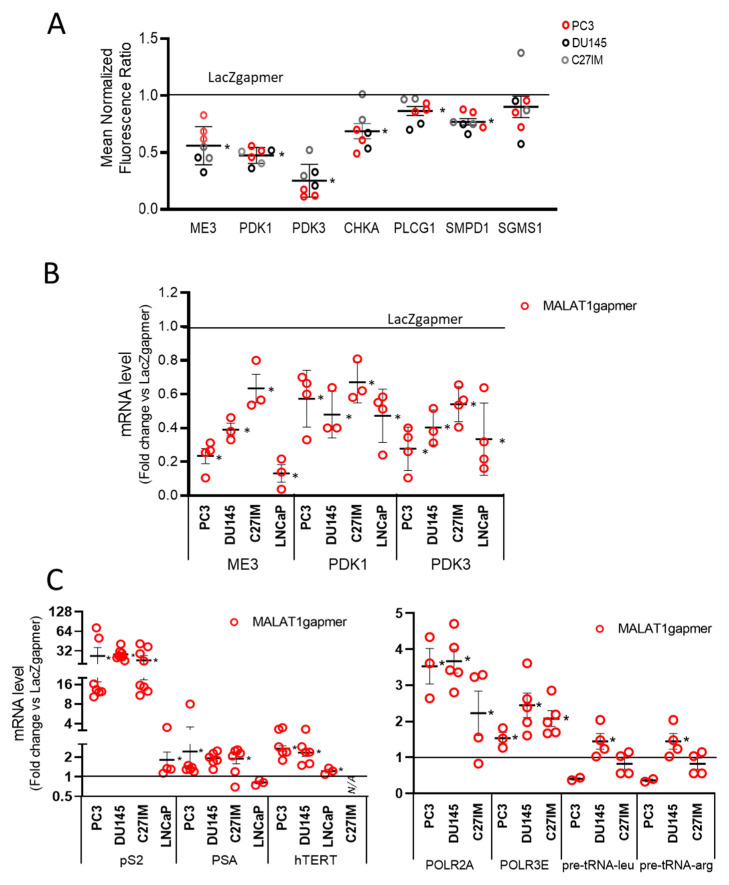
Validation of array data in PCa cells by qRT-PCR. (**A**) Differential expression of genes as emerging from transcriptomic analysis before/after MALAT1 depletion in C27IM, DU145, and PC3 cells. Transcript levels of ME3, PDK3, PDK1, and PCho-related genes (choline kinase A (CHKA), Phospholipase C Gamma 1 (PLGC1), sphingomyelin phosphodiesterase 1 (SMPD1), and sphingomyelin synthase 1 (SGMS1)) are expressed as fold change vs. LacZgapmer (mean ± SEM are showed). (**B**,**C**) mRNA expression level of those genes emerging as differentially modulated were validated in different PCa cells (PC3, DU145, C27IM, and LNCaP) transfected with specific gapmer for MALAT1 or LacZ. Results are plotted as fold induction vs. LacZgapmer (placed to 1 and depicted as a black line). Individual values (*n* = 3–8) with mean ± SEM are showed. Non-parametric paired two-tailed Student’s *t*-test determined statistical significance. * *p* ≤ 0.05 MALAT1gapmer vs. LacZgapmer. * *p* < 0.05 vs. gapmerLacZ.

Of interest, the agilent arrays also showed a strong downregulation of a subset of genes belonging to the phosphatidylcholine pathways such as choline kinase A (CHKA), phospholipase C gamma 1 (PLCG1), sphingomyelin phosphodiesterase 1 (SMPD1), and sphingomyelin synthase 1 (SGMS1) (Figure 3A). The control of this family of genes is often lost in PCa [29]. Figure 3B shows the qPCR validation of several downregulated genes, including ME3, PDK1, and PDK3, which changed significantly upon MALAT1 targeting. In the same condition, we found an upregulated expression of several hormone-responsive genes, including Trefoil factor 1 (TFF1 or pS2), *Prostate-specific antigen (*PSA), and human telomerase reverse transcriptase (hTERT), as well as that of RNA polymerase II subunit A and 3 (POLR2A), POLR3), and transfer ribonucleic acid (tRNAs) (Figure 3C).

#### 2.1.3. Expression of the Electron Transport-Chain Protein Complexes and Citrate Production in MALAT1-Depleted Metastatic Prostate Cancer Cell Lines

**Figure 4:**
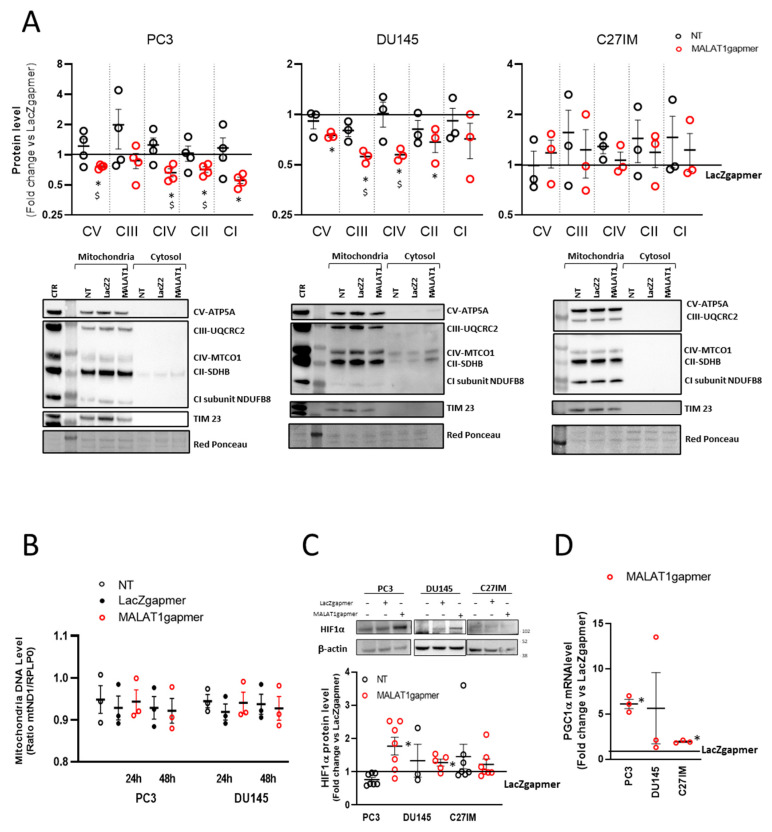
Metabolic alteration after MALAT1 depletion. (**A**) Western blotting analysis of OXPHOS complex subunit expression determined by using total OXPHOS antibody cocktail in PC3 (left), DU145 (middle), and C27IM (right) cells’ mitochondrial fractions upon MALAT1 depletion. Upper panels: Densitometric analysis plotted as fold change vs. LacZgapmer after normalization to loading control red ponceau. Lower panel: Representative western blotting experiments. Uncropped Western Blots of Figure 4A are available in Figure S9 (**B**) qRT quantification of mitochondrial DNA level in PC3 and DU145 cells at 24 h or 48 h after transfection with specific (MALAT1), control (LacZ), or no (NT) gapmer. Data are expressed as ratio mtND1 vs. RPLP0 level. (**C**) Representative western blot and densitometry analysis for HIF1α assessed in 3 different PCa cells (PC3, DU145, and C27IM) not transfected or transfected with MALAT1 or LacZgapmers. β-actin was used as a loading control. Uncropped Western Blots of Figure 4C are available in Figure S10 (**D**) qRT expression of PGC1alpha mRNA in different PCa cells (PC3, DU145, and C27IM) interfered with MALAT1 or LacZgapmers. Results are plotted as fold induction vs. LacZgapmer taken as reference value 1 (straight black line). Individual values (*n* = 3–8) with mean ± SEM are shown. Non-parametric paired two-tailed Student’s *t*-test determined statistical significance. * *p* ≤ 0.05 MALAT1gapmer vs. LacZgapmer. ^$^ *p* ≤ 0.05 MALAT1gapmer vs. NT.

In cells depleted of MALAT1, transcriptome analysis suggested that mitochondrial function was compromised. This observation prompted us to evaluate the OXPHOS subunits’ expression in the mitochondrial fraction prepared from three PCa cell lines and analyzed by using a validated OXPHOS antibody cocktail. The cocktail contains antibodies for nuclear-encoded subunits of complex V (ATP5A), III (UQCRC2), II (SDHB), I (NDUF8), and the mitochondrially encoded subunit of complex IV (CIV-MTCO1). The samples were also probed with TIM 23 antibody as quality control for lysates of mitochondrial origin. Figure 4A shows that as early as 24 h from MALAT1 depletion, some subunits’ protein levels significantly decreased in PC3 and DU145 cells. Specifically, the MTCO1 and the SDHB subunits of complex II were downregulated in both metastatic cell lines, whereas the other complexes appeared differentially regulated: CI-NDFB8 being downregulated in PC3, while CV-ATP5A and CIII-UQCRC2 were reduced in DU145 cells. On the contrary, primary-tumor derived cell line C27IM cells did not show any change in the OXPHOS components.

To investigate whether these changes in the respiratory chain complexes were associated with reduced mitochondrial mass, the intracellular level of mitochondrial DNA was evaluated after MALAT1 depletion and in control conditions. Figure 4B shows that in all conditions tested, the mitochondrial DNA content showed no significant variation than controls. The intracellular concentrations of ATP and ADP were measured in protein-free cell extracts to evaluate the consequences of the changes mentioned above. Table S3 shows that, after MALAT1 depletion, no changes were detected in the ATP and ADP content or the ATP/ADP ratio in PC3 cells, suggesting that, at the given time point, the energy production homeostasis was not affected by MALAT1 targeting.

Next, we evaluated the possibility that the altered mitochondrial pattern might be associated with an intracellular hypoxic environment. Consistently, the HIF1-alpha protein level was also induced in PC3 and DU145, suggesting that the metabolic environment of MALAT1-depleted PCa cells reflected a pseudo-hypoxic condition (Figure 4C). No stabilization was appreciated instead in C27IM cells. In line with this, we measured in three PCa cell lines, before/after MALAT1 silencing, the mRNA level of the peroxisome proliferator-activated receptor-gamma coactivator (PGC)-1alpha, a member of a family of transcription coactivators that plays a central role in the regulation of cellular energy metabolism [30]. As shown in Figure 4D, PGC1 alpha mRNA significantly increased over control in PC3 and C27IM, whereas in DU145 it did not change significantly.

#### 2.1.4. MALAT1 Knockdown Compromises the TCA Cycle

**Figure 5:**
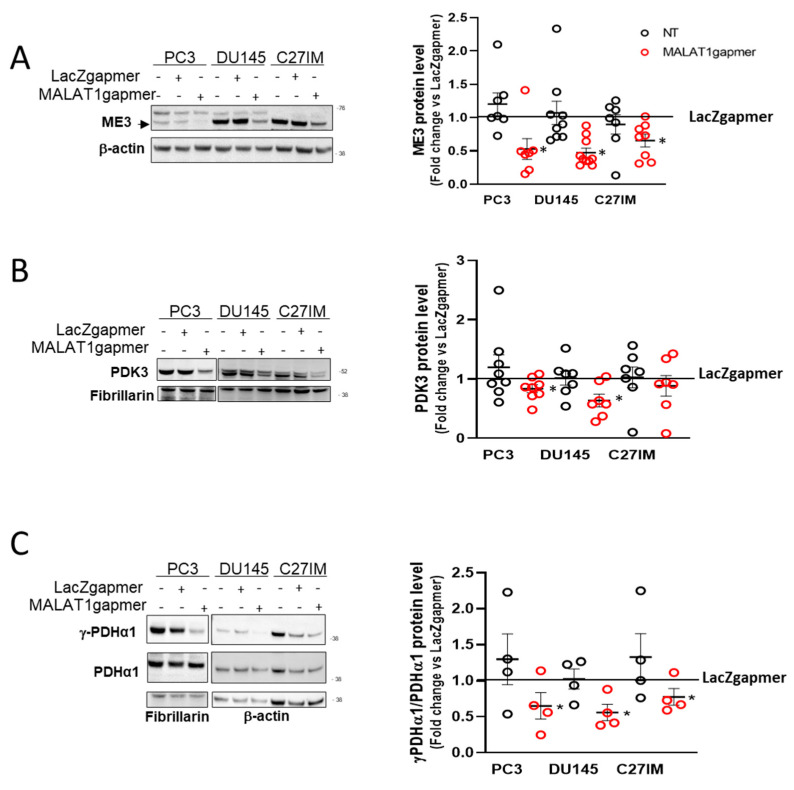
Validation of PCa cells array data set. (**A**–**C**) Representative western blot and densitometry analysis for specific metabolic enzymes assessed in 3 different PCa cells (PC3, DU145, and C27IM) not transfected or transfected with specific gapmers for MALAT1 or LacZ: ME3 ((**A**), black arrow indicates specific signal), PDK3 (**B**), γ-PDHα1, and total PDHα1 (**C**). β-actin or fibrillarin were used as a loading control. ME3 and PDK3 protein levels were normalized versus β-actin and fibrillarin, respectively; γ-PDHα1 versus total PDHα1. Results are plotted as fold change vs. LacZgapmer taken as reference value 1 (straight black line). Individual values (*n* = 4 to 8) with mean ± SEM are shown. Non-parametric paired two-tailed Student’s *t*-test determined statistical significance. * *p* ≤ 0.05 MALAT1gapmer vs. LacZgapmer. Uncropped Western Blots of Figure 5 are available in Figures S11–S13.

The increased glycolytic rate and the impairment of the mitochondrial OXPHOS prompted us to explore further the effect of MALAT1 depletion on the pyruvate conversion and the TCA cycle functionality. To do so, we determined the protein expression of those genes encoding essential mitochondrial enzymes and showing a substantial downregulation at the mRNA level after MALAT1 depletion. Figure 5A–C shows that ME3, PDK3, and the phosphorylated form of PDHA1 were significantly reduced after MALAT1 targeting. These results suggested that MALAT1 depletion might render pyruvate conversion into acetyl-CoA more efficient through the downregulation of PDK3 and an increase in the pyruvate activity dehydrogenase complex as determined by the decrease in the phosphorylation state of the PDHA1 subunit. In this condition, notwithstanding the higher amount of acetyl groups supplied by PDHA1, the TCA cycle functions seem reduced after MALAT1 depletion.

Indeed, the decreased succinate dehydrogenase activity and IDH2 protein level (Figure S3B,C) suggest a deceleration of the TCA cycle in PC3 cells after MALAT1 targeting. This observation is consistent with an imbalance in the pyruvate metabolism, as suggested by the decrease of the ME3 gene and protein expression paralleled by increased NADPH concentration, as shown in Table S4. Further, Figure S4A–C shows that in PC3 cells, during a time course from 1 to 72 h following gapmers transfection, the downregulation of endogenous ME3, PDK1, and PDK3 occurred as early as 4 h from transfection. On the other end, among the upregulated genes relevant for the disease, the time course revealed a delayed and differential induction of mRNAs for pS2, hTERT, and PSA, as well as Cadherin 1 (CDH1) upon MALAT1 targeting, as shown in Figures S5 and S6.

**Figure 6:**
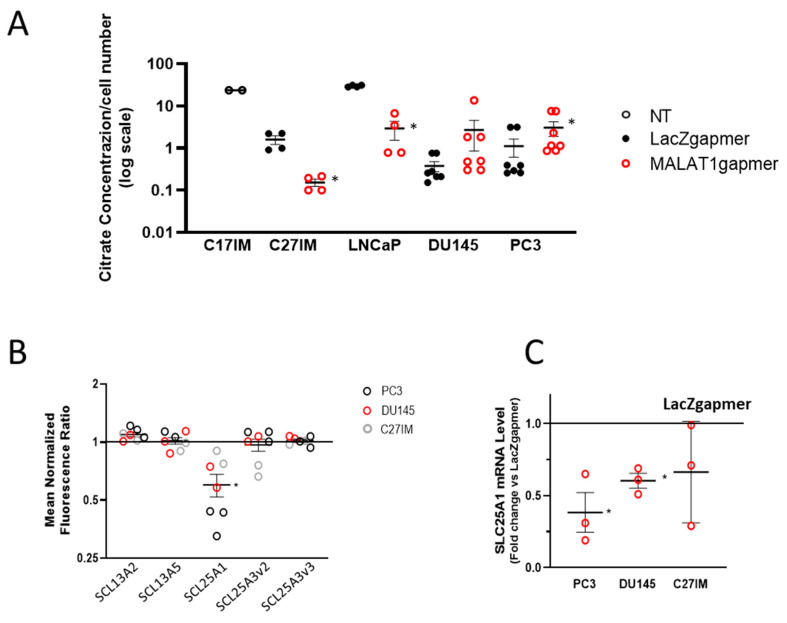
Evaluation of MALAT1 targeting on citrate production and expression of solute carrier transporters. (**A**) Citrate concentration analysis by colorimetric assay in the extracellular medium and normalized to cell number (10^6^ cells) of normal/benign hyperplastic lesion-derived cells (C17IM) used as reference control or PCa cells (C27IM, LNCaP, DU145, and PC3) analyzed before/after MALAT1 targeting. Results are plotted as individual values (*n* = 4–7) with mean ± SEM. (**B**) Citrate transporters genes emerging from transcriptomic analysis before/after MALAT1 depletion in C27IM, DU145, and PC3 cells. Transcript levels of SLC13A2, SLC13A5, SLC25A1, SLC13A3 variant 2 (SCL13A3v2), and SLC13A3 variant 3 (SCL13A3v3) are expressed as fold change vs. LacZgapmer (mean ± SEM are showed). (**C**) SLC25A1 expression level validated in different PCa cells (PC3, DU145, and C27IM) transfected with specific gapmer for MALAT1 or LacZ. Results are plotted as fold change vs. LacZgapmer (placed to 1 and depicted as a black line). Individual values (*n* = 3) with mean ± SEM are shown. Non-parametric paired two-tailed Student’s *t*-test determined statistical significance. * *p* ≤ 0.05 MALAT1gapmer vs. LacZgapmer.

Given the relevance of citrate metabolism in the homeostasis of prostate epithelial cells, we also measured citrate production in the extracellular medium of PC3, DU145, LNCaP, C27IM, and C17IM cells (Figure 6A) before/after MALAT1 targeting. As expected, basal citrate levels were elevated in the C17IM cell line, established from a benign prostate hyperplasia BPH lesion [31]. Interestingly, citrate production was detectable in C27IM and LNCaP in the basal condition and significantly decreased after MALAT1 targeting. In contrast, in metastatic PC3 and DU145 cells exhibiting low basal levels of citrate in the extracellular medium, citrate secretion was significantly recovered or showed a trend towards increasing, respectively. In parallel, we checked the expression of solute carrier transporters present in the agilent platform (Figure 6B), and, in all prostate cancer cell lines analyzed upon MALAT1 depletion, we found a statistically significant downmodulation exclusively of SLC25A1, a mitochondrial citrate transporter mediating the exchange of mitochondrial citrate with cytosolic malate [32,33,34]. Downmodulation of SLC25A1 was confirmed by qPCR in PC3 and DU145 cells (Figure 6C).

#### 2.1.5. Silencing ME3 Increases Lactate Production

**Figure 7:**
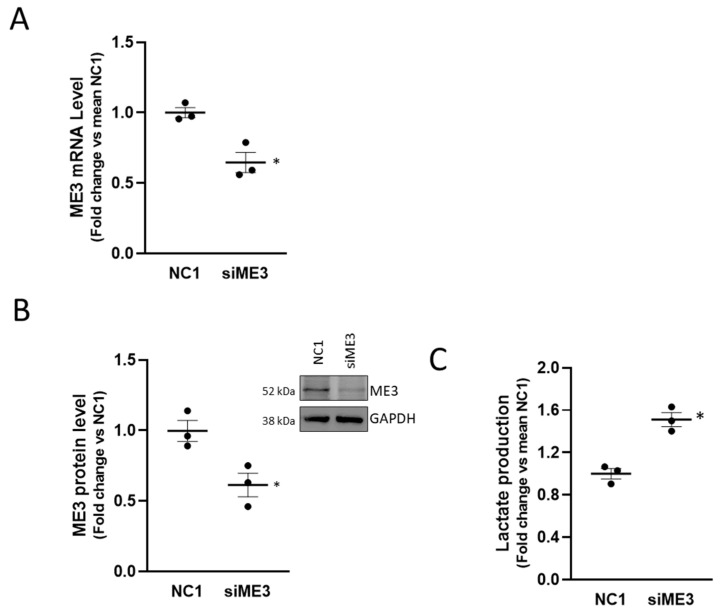
Effect of ME3 silencing on lactate production: (**A**) qPCR determination of ME3 mRNA level after 48 h of ME3 silencing (siME3) compared to control (NC1). (**B**) Representative western blot of ME3 after 48 h of ME3 silencing in PC3 cells (right panel); densitometric analysis of ME3, GAPDH was used as a loading control. Uncropped Western Blots of Figure 7B are available in Figure S14. (**C**) Lactate concentration analysis by colorimetric assay in PC3 cells after ME3 silencing collected at 48 h after transfection. Results are plotted as fold change vs. mean NC1. Individual values (*n* = 3) with mean ± SEM are shown. Non-parametric paired two-tailed Student’s *t*-test determined statistical significance. * *p* ≤ 0.05 MALAT1gapmer vs. LacZgapmer.

The malic enzyme catalyzes the oxidative decarboxylation of malate to pyruvate using either NAD+ or NADP+ as a cofactor. Mammalian tissues contain three distinct isoforms of malic enzyme: A cytosolic NADP(+)-dependent isoform, a mitochondrial NADP(+)-dependent isoform, and a mitochondrial NAD(+)-dependent isoform. The *ME3* gene encodes a mitochondrial NADP(+)-dependent isoform, which catalyzes the decarboxylation of malate to pyruvate, contributing indirectly to the TCA cycle via the malate/pyruvate cycle [35]. Its role in cancer, however, is not fully understood. In pancreatic cancer, ME3 promotes proliferation, epithelial to mesenchymal transition, and aggressiveness [36]. To assign a role to ME3 in the metabolic perturbation mediated by MALAT1, we interfered with ME3 by siRNA in PC3 cells obtaining a 50% protein reduction (Figure 7A,B) and evaluated the consequences of ME3 silencing in terms of lactate production (Figure 7C). Of interest, we appreciated a modest but reproducible increase in lactate accumulation (~1.5 fold over control), partially mimicking the effect observed upon MALAT1 targeting. This finding revealed an unprecedented functional relationship between MALAT1 and the ME3 that might represent a crucial transitional element between oxidative phosphorylation and glycolysis toward the Warburg effect.

#### 2.1.6. Consequences of MALAT1 Depletion in Human PCa-Derived Organotypic Slice Cultures (OSCs)

**Figure 8:**
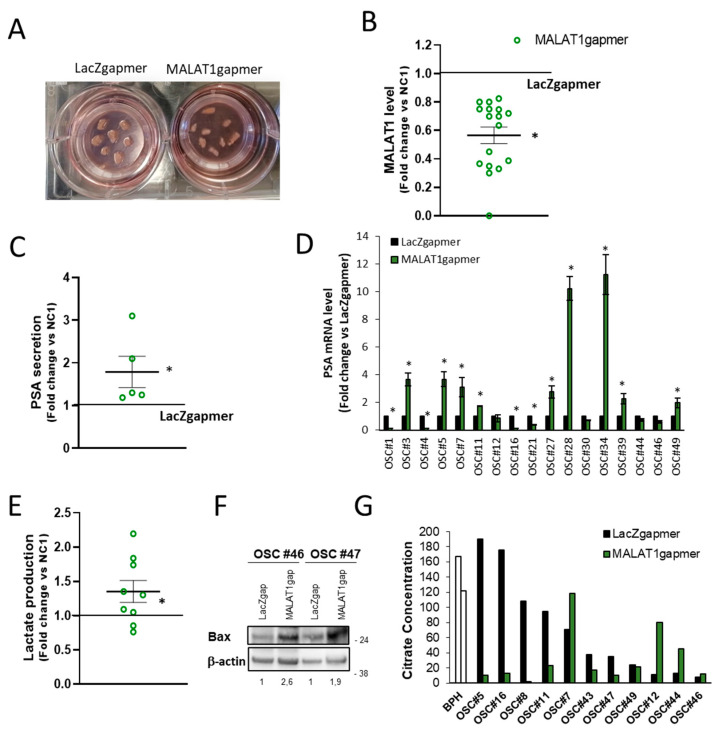
Organotypic slice cultures of prostate tumors and in vivo effects of MALAT1 depletion. (**A**) Image of representative human PCa organotypic slice cultures (OSCs) obtained from intra-operatory prostate cancer specimens transfected with specific (MALAT1) or control (LacZ) gapmer. (**B**) MALAT1 expression level was quantified by qRT-PCR in OSCs (*n* = 17) at 72 h after transfection with specific MALAT1gapmer or LacZgapmer. (**C**) PSA concentration measured in the extracellular medium of OSCs (*n* = 5) at 24h after transfection with MALAT1gapmer or LacZgapmer. (**D**) PSA mRNAs in 17 OSCs as in (**B**,**E**). Lactate concentrations analysis assessed using a colorimetric assay in the supernatant of OSCs (*n* = 9) at 24 h after transfection with MALAT1 silencing. Results are plotted as fold change vs. LacZgapmer (placed to 1 and depicted as a black line). Individual values (*n* = 3) with mean ± SEM are showed (green circles for MALAT1 gapmer). Non-parametric paired two-tailed Student’s *t*-test determined statistical significance. * *p* ≤ 0.05 MALAT1gapmer vs. LacZgapmer. (**F**) Representative western blot of BCL2 Associated X (BAX) after MALAT1 silencing in OSCs β-actin was used as a loading control. Numbers represent the fold induction vs. LacZgapmer normalized to loading control. A molecular weight marker is indicated. Uncropped Western Blots of Figure 8F are available in Figure S15 (**G**) Citrate concentrations analysis by colorimetric assay in the cell culture supernatant of normal/benign hyperplastic prostate (BPH) and in several PCa-derived OSC before and after MALAT1gapmer collected after 48 h of interference. Citrate concentration was normalized to relative βactin level. OSC #5, #16, #11, #7, #49, #12, #44, #46 exhibited an efficient MALAT1 depletion. Results are plotted mean ± SEM.

During the progress of this study, we developed an ex vivo human prostate cancer model based on organotypic slice cultures (OSCs). OSCs recapitulate the distinctive characteristics of the tissue of origin (including androgen/estrogen responsiveness of known targets such as PSA or pS2) with the advantage that can be cultured and genetically manipulated, representing a unique source of relevant translational information at a single patient level [37,38] to investigate PCa biology (Figure 8A). We collected a series of OSCs obtained from a cohort of 50 PCa patients diagnosed with localized disease and undergoing surgery (Table 1).

The OSCs in which parameters such as morphology, immunophenotype, and hormone responsiveness were not appropriate [37,38] or the amount of tumor tissue was ≤60%, as estimated by the pathologist on the original histopathological slide, were excluded from the study. Of the remaining 40, qPCR’s MALAT1 basal levels presented a broad distribution (Figure S8A), and transfection with MALAT1 gapmer resulted in a significant depletion of MALAT1 in 17 out of 40 (Figure 8B and Figure S8B). This cohort of 17 OSCs was further analyzed. Of interest, increased PSA secretion in the extracellular medium of a subset of OSCs (Figure 8C) was paralleled by an increased PSA mRNA in 8 out of 17 efficiently MALAT1-silenced OSCs (Figure 8D). This finding appears in agreement with the increased expression of PSA mRNA observed in cell lines upon MALAT1 depletion (Figure 3C and Figure S4, Table S5, and [37]). In the same condition, lactate increased in the extracellular medium (Figure 8E) paralleled by the upregulation of the apoptosis regulator Bcl-2-associated X protein Bax (Figure 8F). Citrate production was also measured in several OSCs (Figure 8G). As expected, basal citrate concentration in the extracellular medium of BPH-derived OSCs was elevated, ≈150 mg/L. Most OSCs derived from primary PCa (8/11) exhibited a variable but a relatively high citrate (ranging from 190 to 40 mg/L) except for OSC#12, OSC#44, and OSC#46, whose production was under the detection threshold ≤10 mg/L. Of note, MALAT1 targeting in OSCs caused abrogation of citrate production in 6/11 or no change in 2/11 when OSCs retained the ability to produce citrate, and an increase in 3/11, namely OSC#7, OSC#12, and OSC#44, in which MALAT1 silencing appears to restore citrate secretion as observed in PC3 cells (Figure 6A). Overall, the detectable basal level of citrate agrees with values found in BPH or primary tumor-derived cell lines (C17IM or C27IM) and LNCaP, yet representative of hormone-dependent PCa.

#### 2.1.7. Validation of Arrays Data Set in OSCs

**Figure 9:**
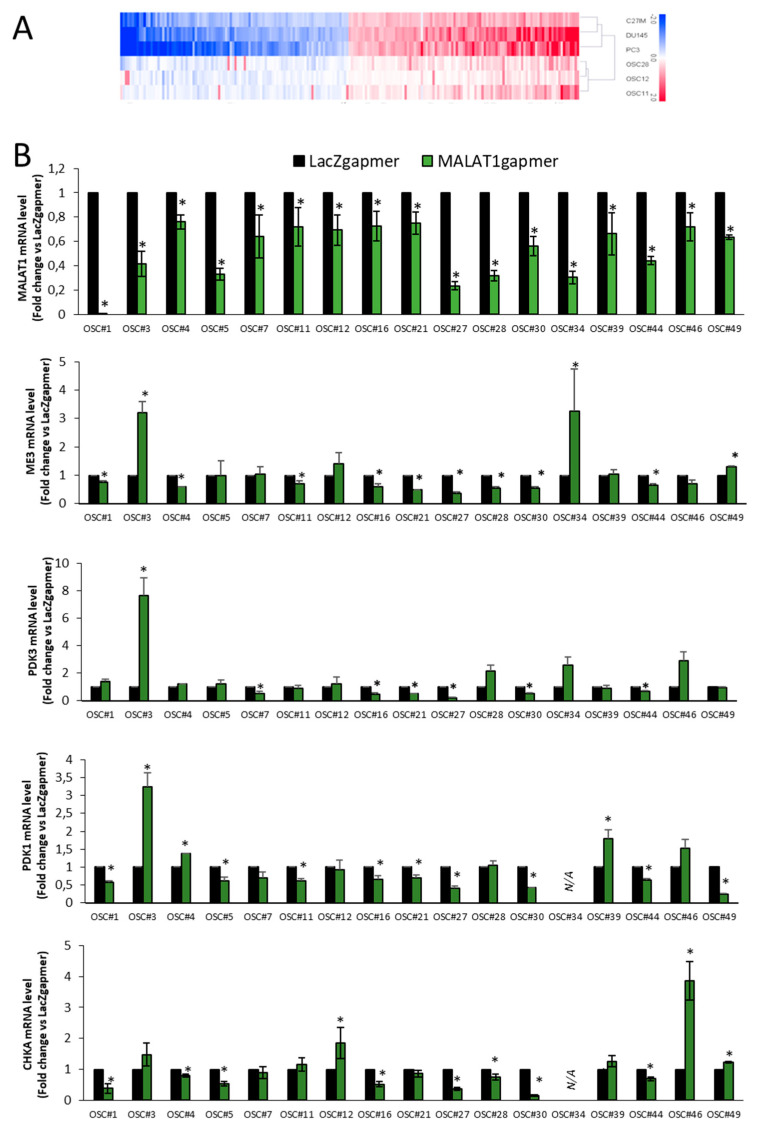
Validation of array data in organotypic slice cultures (OSCs). (**A**) Heatmap of concordant genes (*n* = 200) sharing the same regulation after MALAT1 depletion in PCa cells (C27IM, DU145, and PC3) and OSC#28-12-11. Mean log2FC are represented with red stripes for upregulated genes and blue for downregulated genes. (**B**) Validation of MALAT1, ME3, PDK3, and PDK1 and CHKA as representative genes emerged from array analysis before (white bars) and after (green bars) MALAT1 depletion in different OSCs. Data are expressed as fold change vs. LacZgapmer (mean ± SEM). * *p* < 0.05 MALAT1gapmer vs. LacZgapmer.

Whole transcriptome analysis was performed in three primary-tumor-derived OSCs obtained from fresh PCa explants before/after MALAT1 targeting. The selection of these OSCs was based on biochemical/local recurrence in donor-patients, as indicated in Table 1 (OSC#11 and OSC#28) associated with recurrence, while OSC#12 was not). LacZgapmer was used as a control reference. Figure 9A depicts a heatmap representing the expression profile of about 200 genes co-regulated after MALAT1 targeting in C27IM, DU145, and PC3, and OSC#28, #12, #11. Among the upregulated, there were members of the poly (ADP-ribose) polymerases (PARPs, specifically PARP10, 14, 8) family that share the ability to catalyze the transfer of ADP-ribose to target proteins (poly ADP-ribosylation) [39]. Conversely, among the co-repressed genes, we found again ME3 and SMPD1, which appear to be relevant MALAT1-dependent genes in vitro and in vivo experimental models.

Validation of ME3, PDK3, PDK1, and CHKA repressed after MALAT1 depletion was performed in 17 OSCs by qPCR (Figure 9B). These genes were repressed in 9/17 (ME3), 6/17 (PDK3), 9/16 (PDK1), and 9/16 (CHKA) OSCs. Of note, OSC#3 and OSC#34 exhibited upregulation of ME3, PDK3, and PDK1, while OSC#46 of CHKA, despite a significant MALAT1 targeting. This phenomenon might reflect the complex heterogeneity of the OSCs’ microenvironment or differences between individual patients. Variation in the expression between different OSCs of MALAT1, ME3, PDK3, PDK1, and CHKA is shown in Figure S8B–F.

#### 2.1.8. Partial Least Squares Discriminant Analysis (PLS_DA)

Based on our experimental evidence, we attempted to develop patient-specific optimized algorithms to predict patient outcome. Table S5 reports mean and standard deviation and median values and ranges of the quantitative variables collected and numbers and percentages of the dichotomous variables for the entire sample (*n* = 40) divided into the non-recurrent or recurrent group (*n* = 28 and *n* = 12, respectively). As expected, the classical variables (see Methods), precisely International Society of Urological Pathology (ISUP) grading of prostate cancer, “pathological stage” and “adverse clinical features,” resulted significantly different in the two groups. Specifically, values for ISUP score were 2.25 ± 0.80 vs. 3.1 ± 0.70 (median: 2 vs. 3); values for “pathological stage” were 2.86 ± 0.71 vs. 3.92 ± 0.90, and values for “adverse clinical features” were 1.00 ± 1.18 vs. 2.83 ± 1.67 in patients who did not experience disease recurrence vs. patients with recurrence, respectively (*p* < 0.05). “Extracapsular extension” and “seminal vesicles invasion” resulted significantly associated with the disease recurrence (*p* = 0.005 for both tests). For the “extracapsular extension” variable, 75% of patients with recurrence had a positive response versus only 25% of patients without recurrence. For the “seminal vesicles invasion” variable, 33% vs. 0% of positive values were recorded in patients with and without recurrence, respectively.

**Figure 10:**
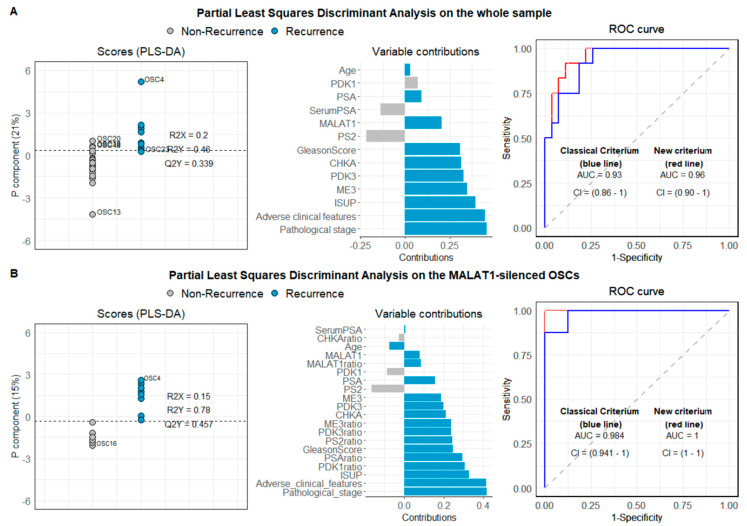
Partial least squares discriminant analysis (PLS-DA) on PCa-derived OSCs. PLS-DA was conducted on the whole sample (**A**) and MALAT1-silenced OSCs (**B**). The left panel reports the scores from the single component PLS-DA model. The separation threshold (dashed line) derives from the Youden criteria. The middle panel reports the variable loadings on the model component. Colors indicate the class (non-recurrent vs. recurrent patients) for which each variable assumes the maximal mean value. The right panel reports the ROC curves derived from the PLS-DA models for both the model, including the gene expression (new criterium) and the model including only the variables collected in the standard clinical practice (classical criterium).

We then evaluated the basal expression of candidate genes emerging from the transcriptomic analysis in our whole sample OSCs (*n* = 40) by qPCR, and, when considered a single variable, no significant difference was found between recurrent and disease-free patients. Next, we attempted to define an algorithm combining classical histopathological parameters and variables identified by gene profiling and subsequently validated by qPCR, named new criterium. For the new criterium, the optimum model resulted in a single component model; 21% of the variable variance was captured by the component with a Q2 value of 0.40, which, compared with the value distribution obtained after random permutations, led to a *p* of 0.001. Receiver operating characteristic—(ROC) area under curve (AUC) computed to assess the partial least squares discriminant analysis (PLS-DA) classification power was equal to 0.96 (CI 95% = 0.90–1, the red line in Figure 10A, right panel). According to the Youden criterium [40] the model showed a good classification performance with a misclassification error rate of 10%. Figure 9A (left panel) reports the OSC scores and the separation threshold derived from the Youden criterium. The middle panel displays the loading weights, where colors indicate the class (non-recurrent vs. recurrent patients) for which the selected variable assumes the maximal mean value. Recurrent patients showed high values for all the variables associated with tumor severity and exhibited higher expression levels for ME3, PDK3, CHKA, and MALAT1. The component was highly and positively correlated with the pathological stage (0.71), ISUP score (0.61), adverse clinical features (0.69), ME3 (0.54), PDK3 (0.50), and CHKA (0.49), and negatively correlated with PS2 (−0.34) and serum PSA (−0.21). When only tumor-related variables, along with the serum PSA and the age (classical criterium), were used in a PLS-DA, the final model performed worse. The Q2 value resulted in almost the same (0.33, *p* = 0.001), but the ROC AUC was 0.93 (CI 95% = 0.86–1, the blue line in Figure 10A, right panel) and the classification error was 18% (three patients more were misclassified).

The same analysis was also performed considering only the OSCs in which the MALAT1 silencing procedure resulted to be efficient (*n* = 17 with at least 17% of MALAT1 depletion). Seventeen OSCs satisfied the criterium, and sixteen entered the PLS-DA analysis due to missing values on PDK1 and CHKA variables for one OSC. For this subsample, MALAT1 resulted significantly reduced (*p* < 0.0001), while PS2 was significantly augmented (*p* = 0.04). Eight out of sixteen patients experienced disease recurrence. Table S6 reports the subsample’s descriptive variable, along with *t*-tests and Mann–Whitney U test for difference testing between recurrent and non-recurrent (*n* = 8 and *n* = 9, respectively). Results from the PLS-DA are summarized in Figure 10B. In this case, the model’s goodness resulted in a single component model, with 15% of the variable variance captured by the component. The model combining classical histopathological parameters and basal level of genes emerged by gene profiling, as well as their modulation after MALAT1 silencing (new criterium bis), showed an excellent classification performance with a misclassification error rate of 0% (according to the Youden criterium) and a Q2 value of 0.42, which, compared with the value distribution obtained after random permutations, led to a *p* of 0.01. On the contrary, the classical model based on the traditional parameters (pathological stage and adverse clinical features) led to a low classification error (6.2%). In this case, OSC49 was not correctly classified.

## 3. Discussion

Among males, prostate cancer was one of the three most common neoplasias in 2019 in the USA (3,650,030), followed by colorectal cancer (776,120) and melanoma (684,470), and it is the second most frequent cancer for incidence after lung cancer [1,2]. Of note, in PCa, the rate of biochemical recurrence is elevated even though initial radical prostatectomy is considered a definitive treatment for the localized disease [41]. Indeed, approximately 30% of patients experience recurrence after primary therapy and might develop castration-resistant prostate cancer for which current anticancer treatments are not resolutive [42]. Therefore, biochemical recurrence portends a lower survival following surgery. In this light, identifying possible new targets predicting PCa progression or outcome holds excellent promises for aggressive/metastatic PCa management. In this direction, the study of long non-coding RNAs’ regulation and function might open new avenues for developing more effective diagnostic, predictive, and therapeutic tools.

Among lncRNAs, MALAT1 is probably the best characterized in cancer biology. In fact, in colorectal cancer, high levels of MALAT1 have been associated with increased proliferation and migration [20], whereas in esophageal cancer, it promotes epithelial-to-mesenchymal transition through the modulation of the Notch pathway [22]. In line with these findings, we observed an increase of CDH1 expression in all 4 PCa cell lines analyzed upon MALAT1 targeting (Supplemental Figure S6). Notably, high levels of MALAT1 confer resistance to pharmacological treatment, such as cisplatin in lung cancer [24], while, in ovarian cancer MALAT1 interferes with the Notch pathway, conferring chemo-resistance [23]. In PCa, earlier findings showed that MALAT1 is highly expressed and positively related to disease severity and poor prognosis [43]. Moreover, MALAT1 knockdown hampered PCa tumorigenesis and progression, as demonstrated by the reduction of cell proliferative, migratory, and invasive capacities in PCa cells and xenografts following MALAT1 depletion [44], hinting at the potential value of MALAT1 in PCa diagnosis [45], prognosis prediction of, and treatment outcome.

Interestingly, MALAT1 knockdown affected the expression of NEAT1, located less than 70 kb from MALAT1 on human chromosome 1, and on that of the MALAT1 antisense transcript TALAM [46,47]. Hence, the effects reported in this work suggest to be a consequence of the remodeling of the entire genomic region encompassing MALAT1, NEAT1, and TALAM. However, further work is required to address the precise role of each of these factors in PCa. Nevertheless, the sole direct targeting of MALAT1 was sufficient to significantly remodel metabolism eliciting reduction in cell growth and survival either in cultured cells as well as in primary tissue slice cultures (Figure S1 and Figure 8C–G). Specifically, the effect of MALAT1 deprivation was preceded by a metabolic reprogramming associated with an increased glycolytic rate and functional lactate production, reducing the TCA cycle rate’s importance. This effect partly recapitulated healthy prostate cells’ metabolic landscape, in which glycolysis and lactate production are associated with a hypo-functioning TCA cycle [45] (Figures S1, S3 and Figure 4). To interpret this observation, we should consider that the allosteric modulation of phosphofructokinase-1 (PFK1) is one of the mechanisms utilized by mammalian cells to control the glycolytic pathway’ rate through positive and negative involvement of allosteric modulators. Besides them, citrate plays a fundamental role in the downregulation of the activity of PFK-1, acting as a negative allosteric modulator. In this scenario, the allosteric modulation of PFK-1 in normal epithelial cells is still controversial and debated in literature: On the one hand, the high efficiency of citrate transporter of excretion in the extracellular milieu might favor the removal of the allosteric inhibition of PFK, thereby increasing the glycolytic rate [6].

On the other hand, the intracellular concentration of citrate in normal human prostate cells is estimated to be in the range of 1.0–3.0 mM, values sufficient to inhibit PKF-1 [48]. This observation suggests that some peculiar relationships associated with PFK and glycolysis regulation must exist in prostate cells. In this context, more investigations are needed to clarify the link between citrate and the regulation of the glycolytic rate via PFK-1 allosteric modulation.

Herein, we observed that the gapmerization of MALAT1 reversed metabolism in PCa cells and tumor tissue, fostering the partial recovery of glycolytic metabolism. This phenomenon was characterized by the marked downregulation of metabolic enzymes associated with aerobic glycolysis transition to mitochondrial metabolism and significant lactate production due to the glycolytic switch. In this regard, transcriptomic analysis indicates the downregulation of several crucial components of the metabolic machinery associated with pyruvate transition from the cytoplasm to mitochondria (Figure 2 and Figure 3). Among others, ME3, which converts malate into pyruvate, is one of the genes ranking first in the MALAT1-dependent shared signature obtained by combining transcriptome from 3 PCa cell lines and 3 OSCs and appears consistently decreased in terms of mRNA and protein expression in PCa cells as well as in OSCs (Figure 9). Interestingly, ME3 has been recently reported as contributing to proliferation and aggressiveness in pancreatic cancer via its impact on energy production [36]. Here, we can assume that the ME3 function that participates in converting malate into pyruvate (malate/pyruvate cycle) is impaired due to MALAT1 depletion.

ME3 is an enzyme that uses NADP+ as a cofactor. The finding of an increased NADP+/NADPH ratio in MALAT1-depleted PC3 cells keeps with the decrease of ME3 expression being this enzyme NADP+ dependent (Table S4). The observation that the knockdown of ME3 in PC3 cells (Figure 6) determined lactate accumulation reinforces the hypothesis that ME3 plays a role in the MALAT1-dependent metabolic regulation. Noteworthy, in pancreatic cancer, ME3 expression has been recently found higher in tumors than that in non-tumor tissues, and patients with higher ME3 levels had significantly shorter survival than those with lower levels according to the Badea and The Cancer Genome Atlas (TCGA) databases () [36]. Further, the reduction in PDK3 paralleled by the decrease in phosphorylation of the PDHA1 subunit, observed in MALAT1-depleted PCa cells (Figure 5), suggests a higher efficiency mitochondrial multi enzymatic PDH complex converting pyruvate into acetyl-CoA. On the opposite, the experimental evidence suggests that the acetyl-CoA production could be impaired by the downregulation of the isocitrate dehydrogenase 2 (IDH2) protein [49], an effect paralleled by a decreased activity of succinate dehydrogenase and overall downregulation of electron transport-chain protein complexes ( Figure S2 and Figure 4). Altogether, these observations suggest a reduction in TCA cycle function concurrent with the switch to glycolysis.

In agreement with this scenario, we observed a decrease in NADPH’s intracellular concentration, a fundamental cofactor for a wide range of biosynthetic pathways, particularly fatty acid biosynthesis [50,51].

In line with this finding, the decreased proliferation index of MALAT1-depleted PCa cells might be the consequence of a diminished NADPH supply, slowing down the biosynthetic reactions necessary for cell proliferation. Moreover, the restored citrate excretion phenotype of MALAT-1 depleted PC3 cells can readily contribute to the decreased rate of fatty acid biosynthesis through the high-efficiency citrate excretion via _m_SLC25A1 and _pm_SLC25A1, which can rapidly remove citrate from the cytoplasm. The reduction of the availability of citrate as a substrate for the acetyl-CoA (ACC) enzymatic reaction that produces acetyl-CoA into the cytoplasm (utilized as a precursor for fatty acid biosynthesis) could be responsible for the decreased rate of the aforementioned biosynthetic process and the increased value of the NADP^+^/NADP ratio observed in MALAT-1 depleted PC3 cells.

Of note, in PC3 cells, no changes in ATP concentrations and ATP/ADP ratio were observed compared to controls transfected with LacZgapmer. In this context, the downregulation of MTCO1 and the SDHB subunits of the electron transport chain of complex II, observed after MALAT1 depletion, and the concomitant unaltered concentrations of ATP and the high values of the ATP/ADP ratio strongly suggest no uncoupling between ETC and OXPHOS, therefore maintaining relatively unaltered the mitochondrial phosphorylating capacity of MALAT1 depleted cells. However, the preserved ATP levels might also reflect the inhibition of proliferation, which reduces the energy demand in PCa cells and tissue, preparing for the activation of a cell death program. In line with this, as reported by Matheson et al. [52], in the presence of an impairment in mitochondrial phosphorylating capacity (ATP/ADP ratio), PC3 cells could satisfy their energy demand through glycolysis compensating for ATP production [53]. Further, MALAT1 depletion introduced changes in pyruvate kinase activity coupled with a decrease in the NAD+/NADH ratio, accumulation of extracellular lactate levels, and increased activity of lactate dehydrogenase. Since these changes were not observed in LacZgapmer transfected cells, it is conceivable that in MALAT1-depleted PCa cells, a rapid adaptive response, finalized to enhance glycolysis as one of the primary sources of ATP, might take place at least in the short term. Similarly, an overall increase in the glycolytic rate was observed under neurodegenerations characterized by mitochondrial malfunctioning and energy imbalance [53].

In light of these considerations, we analyzed the extracellular concentration of citrate in cells transfected with the specific MALAT1 gapmers or their controls (LacZgapmer) at 24 h after treatment. We observed a recovery of citrate excretion significant in PC3 and as a trend in DU145 cells where basal citrate levels were initially barely detectable but regained after MALAT1 targeting. This phenomenon was not observed in C27IM or LNCaP cells in which the capacity to produce citrate was abrogated (Figure 6A). Interestingly, a positive effect on citrate synthesis after MALAT1 targeting was observed in 3 out of 11 organotypic slice cultures, namely OSC#7, OSC#12, and OSC#44, which, as PC3 cells, had deficient levels of citrate production before MALAT1 depletion (Figure 8G). In line with this, we found downmodulation of the primary prostatic citrate transporters, SLC25A1 (Figure 6B,C), remarkable since its involvement in cancer therapy resistance and stemness [54,55]. This observation suggests that MALAT1 depletion might contribute to the inactivation of this citrate transporter, detrimental to prostate homeostasis and supporting cancer aggressiveness.

Altogether this evidence strongly suggests that metabolic control is among MALAT1 functions. Indeed, MALAT1 targeting is sufficient to restore (i) aerobic glycolysis in all PCa cells and OSCs and (ii) citrate excretion in PC3 cells and a subset of OSCs. Although these results might be limited by the number of samples evaluated, they might also reflect different tumor progression stages in which PCa cells or tissues retain variable degrees of adaptation to MALAT1 depletion.

The substantial decrease of CHKA, the enzyme catalyzing choline’s conversion to phosphocholine, a metabolic reaction in the Kennedy pathway, is of further interest. CHKA is involved in the synthesis of phospholipids and plays a crucial role in regulating cell proliferation, oncogenic transformation, and human carcinogenesis [29]. Interestingly, CHKA is significantly overexpressed in localized primary and metastatic PCa lesions, where it is an androgen-regulated gene and appears to be an independent predictor of biochemical recurrence-free survival in association with other clinical variables [29]. A decrease in CHKA, as we appreciated upon MALAT1 depletion, has been reported to cause inhibition of growth of PCa cells, human PCa explants, and tumor xenografts [29]. Given the relevance of choline Positron Emission /Tomography- Computed Tomography (PET/CT) in PCa patient stratification concerning lymph node involvement for primary surgery and radiation therapy, our finding derived from a completely original approach appeared to link depletion of MALAT1 to a clinically relevant metabolic feature of advanced/metastatic PCa with a potential impact under the prognostic point of view.

Another accomplishment of the present study is identifying potential novel biomarkers emerging from the bioinformatics analysis of the MALAT1-dependent transcriptome of three well-characterized PCa cell lines with aggressive phenotypes before and after MALAT1 depletion. Those targets have been challenged and validated upon MALAT1 depletion in about 50 human OSCs obtained upon surgery from a cohort of patients with a diagnosis of localized tumors at a different stage (Table 1). In the attempt to build up a model with prognostic relevance, we systematically assessed the association between the levels of PSA, pS2, ME3, PDK1, or PDK3 and CHKA mRNAs before/after MALAT1 depletion with age, serum PSA, histopathological pattern, Gleason score, ISUP score, adverse clinical features, and biochemical/distal recurrence (see Methods, Computations). Using the OSCs which satisfied all the criteria in terms of technically suitability or % of the tumor (*n* = 40), we succeeded in the development of an algorithm virtually capable of optimizing prediction of disease progression (Figure 10) beyond that obtained based on the classical clinical, histopathological parameters, mainly (i) the pathological stage, (ii) the adverse clinical features, as well as (iii) the ISUP score. Analysis of the eligible samples revealed that recurrent patients show high values for all the variables associated with the severity of cancer and exhibit high expression levels for ME3, PDK3, CHKA, and MALAT1 transcripts (Figure 10A). Further, analysis of the same variables taken upon MALAT1 targeting revealed a peculiar modulation of PDK1, ME3, PDK3, and PSA (indicated as the “ratio”) in recurrent patients that might represent a novel prognostic molecular tool. As depicted in Figure 10B, these parameters, together with the pathological stage, ISUP score, and adverse clinical features, showed an excellent classification performance with a misclassification error rate of 0%.

In comparison, the classical model based on the traditional/classical parameters (pathological stage, ISUP score, and adverse clinical features) led to a classification error of 6.2%. In our opinion, this observation, although performed on a limited number of samples, could provide useful information to design innovative ways for the management of prostate cancer patients. Using MALAT1-interfered OSCs, the acquisition of prognostic information based on the molecular biomarkers herein might be rapidly available within one week upon surgery. Moreover, if evaluated at low-risk PCa diagnosis, analysis of MALAT1 knockdown could be used in order to better select active surveillance candidates, in addition to classical or other emergent factors [56].

## 4. Materials and Methods

Antibodies: Antibody to E-cadherin, vimentin, fibrillarin, and HIF1α were as described in [38]. Antibody to androgen receptor as in [37]. Antibodies: β-actin (1:2000, Abcam, ab8227), BAX (1:500, Immunological Sciences, AB-10230), GAPDH (1:600, Abcam, ab9484), IDH2 (1:1000, Bioss bs-3947R), ME3 (1:2000, Abcam, ab172972), PDK1 (1:1000, Abcam, ab110025), PDK3 (1:1000, Abcam, ab 154549),γPDH1α (400 µg/mL, Abcam, ab92696), total PDH1α (1:4000, Abcam, ab168379), TIM23 (1:5000, BD Biosciences, 611223), and total OXPHOS antibody cocktail (1:500, Abcam, ab110413).

Cell cultures and MALAT1 silencing. Prostate cancer cells (C27IM, LNCaP, PC3, and PC3-AR) and MCF7 were cultured as described in [37]. Genetic identity of PC3, DU145, and C27IM cell lines were authenticated by BMR Genomics (Padova, Italy) in July 2019. Immunophenotype characterization of C27IM is shown in Supplemental Figures S7. LNCaP and MCF7 were obtained from the American Type Culture Collection.

All cell lines were routinely screened for mycoplasma contamination by indirect (Hoechst) methods [57]. MALAT1 silencing was achieved by specific gapmers or LacZ control gapmers as described in [37]. Briefly, transfection of lncRNA antisense oligos was obtained by using LNA longRNA GapmeRs (Exiqon, Vedbaek, Denmark) [58] with lipofectamine RNAiMAX (Invitrogen, Carlsbad, CA, USA), in accordance with the manufacturer’s instructions.

Trypan-blue cell viability assay. PC3 cells were plated in equal number in p35 dishes and then transfected. At established time points, specifically 6–24–30–48–54–72–78 h, cells were washed, detached with trypsin, and collected. No harvested products were thrown away at this stage. Cells were centrifuged at 800 rpm for 10 min and then resuspended in 100 μL of PBS. Then, 50 μL were picked up, transferred to a 96-well plate and 50 μL of Trypan blue were added. Cells were well suspended and counted, both dead and alive, in a Burker chamber [59].

Mitochondrial DNA content measurement. Total DNA was isolated from cells using DNeasy Blood and Tissue Kits (Qiagen) as per the manufacturer’s instruction. Next, 30 ng DNA was analyzed by real-time qPCR using SYBR green on 7500 instrument (Applied Biosystems) for mitochondrial encoded gene ND1 and nuclear encoded gene RPLP0, using the followings primers: 

RPLP0: 5′-TCGACAATGGCAGCATCTAC-3’ and 5’-ATCCGTCTCCACAGACAAGG-3′

mtND1: 5′-CCCTAAAACCCGCCACATCT-3′ and 5′-GAGCGATGGTGAGAGCTAAGGT-3′

The relative levels of mitochondrial ND1 DNA and nuclear RPLP0 DNA were used to express mitochondrial DNA content.

RNA extraction and real time qPCR. RNA extraction and real time PCR were performed as in [37] using the following primers:

CHKA 5’-GGTCACTTGGGCCAAAACTC-3’ and 5′-GCCGGCTCGGGATGA-3’;

CS 5′-GGGTGCTGCTCCAGTATTATGG-3′ and 5′-GCTCGTGACACCCCAAACA-3′.

LDHA 5′-CTGGGAGTTCACCCATTAAGCT-3′ and 5′-ACAGGCACACTGGAATCTCCAT-3′;

MCT1 5′-GATTGTTGGTGGCTGCTTGTC-3′ and 5′-GACGTATAGTTGCTGTACGGTGTTACA-3′;

MCT2 5′-GGGACTCTTGGTGCCAACAG-3′ and 5′-GGCATTGGTGGCATTTCTG-3′;

ME3 5′-TGAAGAAGCGCGGATACGA-3′ and 5′-GGCCATCCCCTTGTTGAGA-3′;

NEAT1 5′-TCCAGCTGAAAGTTACAAAAATGC-3′ and 5′-GCTCGCCATGAGGAACACTATAG-3′;

PCAT19 5′-AACAGGGAACCATTGGAGATACTC-3′ and 5′-CAGCTCCATAAAGTCAACTTCAATTC-3′;

PDK1 5′-TTTACCCCCCTATTCAAGTTCATG-3′ and 5′-TCGGTCACTCATCTTCACAGTCA-3′;

PDK2 5′-ACACATCGGCAGCATCGA-3′ and 5′-CCATGTCGTAGGCATCTTTGAC-3′;

PDK3 5′-GCATCTCTTTCCGCATGCTT-3′ and 5′-CAGGATTAGTGTCACCCCCAAA-3′;

PGC1a 5′-CTCCCTTGTATGTGAGATCACGTT-3′ and 5′-CTCGTGCTGATATTCCTCGTAGCT-3′;

POLR2A 5′-AACCAGGATGACCTGACTCACA-3′ and 5′-GCCGCAGCTGATTGTTGAT-3′;

POLR3E 5′-ACACGGAGCTCGGTCAAGTCA-3′ and 5′-CTGGCTGGGTGGCATCA-3′;

TALAM1 5′-GGGTGAAGCAGGACAACCA-3′ and 5′-TGATCTCTGCAAACTGCAACCT-3′.

Primers specific to MALAT1, pS2, PSA, hTERT, GAPDH, and β-actin were as in [37]; those for CDH1 as in [38], those for tRNA-LeutRNA-Arg as in [60].

Determination of lactate levels in extracellular medium. Lactate concentration in the extracellular medium in PC3, PC3-AR, DU145, C27IM, LNCaP, and MCF7 was determined using the Lactate Colorimetric/Fluorimetric Assay Kit (Biovision, K607-100) in accordance with the manufacturer’s instructions. The same number of cells before/after MALAT1 targeting were processed and the concentration (μmol/L) of l-lactate in the extracellular medium was determined as follows:

Lactate concentration (nmol/L) = ((OD_570-OD_(570 corrected)) × Diluition factor)−Intercept)/slope;(1)

where the corrected OD (background subtracted), and slope and intercept were calculated from the standard reference curve.

Only for the PC3 cells was determination of lactate in the extracellular medium carried out also by spectrophotometric analysis using an Agilent 89090A spectrophotometer (Agilent Technologies, Santa Clara, CA, USA), following the method described by [61], reported in details [62]. In all assays, the extracellular lactate concentrations were normalized to the number of cells and expressed as µmol/10^6^ of cells.

Determination of intracellular compounds related to the energy metabolism. Before trypsinization, the culture medium’s aliquots were separated and saved at −80 °C for the subsequent lactate determination. Trypsinized cells were counted and then pelleted. Cells were washed with large volumes of ice-cold PBS, pH 7.4, and centrifuged at 1890× *g* for 10 min at 4 °C to complete removal of the cell culture medium and any compound interfering with the subsequent HPLC determination of intracellular metabolites. Cell pellets were treated to determine intracellular compounds related to energy metabolism described in details elsewhere [63,64]. Briefly, samples were treated with a precipitating solution composed of 75% of HPLC grade CH3CN + 25% of 10 mM pH 7.4 buffered KH2PO4 and then centrifuged at 20,690× *g* for 15 min at 4 °C. The protein-free supernatants were collected and subjected to 2 chloroform washings to eliminate the organic solvent and obtain an upper aqueous phase that was directly injected onto the HPLC. The simultaneous separation and quantification of oxidized and reduced nicotinamide adenine dinucleotide (NAD+, NADH), oxidized and reduced nicotinamide adenine dinucleotide phosphate (NADP+, NADPH), adenosine diphosphate (ADP), and adenosine triphosphate (ATP) were performed in each sample of cell extract, according to an ion-pairing HPLC method previously set up in our laboratories [61,62]. The HPLC apparatus consisted of a SpectraSystem P4000 pump and a highly sensitive UV6000LP diode array detector (Thermo Electron Corporation, Milan, Italy), equipped with a 5-cm light-path flow cell, set up between 200 and 400 nm wavelength for the acquisition of chromatographic runs. Data were acquired and analyzed by the Chrom-Quest software package provided by the HPLC manufacturer. Separation of the various compounds was carried out using a Hypersil 250 × 4.6 mm, 5 µm particle-size column provided with its own guard column. Species identification and quantification in deproteinized samples of cell extracts were determined by matching retention times, peak areas, and absorption spectra of freshly prepared ultrapure standards. The concentrations of metabolites of interest were determined at 260 nm wavelength. All concentration values were normalized to the number of cells and expressed as nmol/10^6^ cells.

Determination of enzymatic activity. In a different set of experiments, cell pellets, obtained as described above, were processed for the determination of the enzymatic activity of pyruvate kinase (PK), lactate dehydrogenase (LDH), and succinate dehydrogenase (SDH) by adding 500 μL/106 cells of a hypotonic buffer containing 50 KCl + 10 mM KH2PO4, pH 7.4. After vigorous vortexing for 60 sec, the cell suspensions were sonicated in an ice bath for three cycles of 20 sec each with 20 sec intervals between each sonication pulse (10 kHz of frequency). Subsequently, samples were centrifuged at 20,690× *g* for 15 min at 4 °C to remove any cell debris. Enzymatic activities were performed spectrophotometrically (using an Agilent 89090A spectrophotometer; Agilent Technologies, Santa Clara, CA, USA) on cell lysates. PK and LDH activities were assayed as previously described [50,51]. Both PK and LDH reaction were monitored spectrophotometrically at 340 nm by following for 10 min NADH oxidation. The millimolar extinction coefficient of 6.3 × 10^−3^ of NADH was used to calculate both PK and LDH activities. SDH was assayed, as described in details elsewhere [51]. The reaction began by the addition of 0.05 mM of 2,6-dichlorophenolindophenol (DCIP) and monitored spectrophotometrically at 600 nm by observing the reduction of DCIP as an artificial electron acceptor. The millimolar extinction coefficient of 12.42 × 10^−3^ of DCIP was used for the calculation. All enzymatic activities were normalized to the number of cells and expressed as mIU/10^6^ of cells.

Gene profiling RNA was extracted from two independent biological replicates of three PCa cell lines (C27IM, DU145, and PC3) and three primary-tumor-derived OSCs with MALAT1 depletion compared to GapLacZ controls. RNA quality and quantity were assessed using Agilent 2100 bioanalyzer (Agilent Technologies) and NanoDrop ND-1000 spectrophotometer (Thermo Fisher Scientific, Waltham, MA, USA), respectively. Gene expression profiling was carried out as described in [65] using the two-color labeling method utilizing Low Input Quick Amp Labeling Kit (Agilent Technologies): Labeling, hybridization, slide washing, and scanning were performed following the manufacturer’s protocols. Briefly, mRNA from 100 ng of totRNA was amplified, labeled with Cy3 or Cy5, and purified with columns. Then 300 ng of differentially labeled specimens were hybridized on the Agilent SurePrint G3 Human Gene Expression v3, 8 × 60K Microarrays. For each cell line biological replicate and each OSC (except for OSC#11 for which a low amount of RNA was available), two independent hybridizations were carried out, with dye-swap labeling. PC3 cells with and without MALAT1 depletion were hybridized three times, with a technical dye-swap replicate for one of the two independent biological replicates. After hybridization, slides were washed and scanned using the G2505C Agilent scanner.

Raw data elaboration was performed with linear models for microarray analysis (LIMMA) within the bioconductor. Raw intensity values were background subtracted (method = normexp, offset = 50) and normalized using the loess method for the within array normalization, and aquantile, for the between array normalization [66]. Results were represented in log2FC for heatmaps and MA-plots and fold change (FC) for dot-plots, where FCs indicate the mean normalized fluorescence in MALAT1-depleted samples vs. controls. Raw and processed data were deposited on the GEO Omnibus database (GSE14*7*566).

Protein extraction and western blot: Total protein extracts were obtained as in [67] and in [38]. In accordance with the manufacturer’s instructions for the Mitochondria Isolation Kit for Cultured Cells (Thermo Scientific, Waltham, MA, USA), mitochondrial preparation was performed. Mitochondria were lysed in buffer containing 50mM Tris-HCl (pH 8), 150 mM NaCl, 1% IGEPAL CA-630, 0.5% DOC, 0.1% SDS, and supplemented with 1mM phenylmethylsulfonyl fluoride (PMSF and protease inhibitor mix. Western blot assay was performed used 30 µg of total extract, or 5µg of mitochondrial extract, and proteins were solved by SDS-PAGE using 4–12% gradient Invitrogen Precast Gel (NuPage and MES buffer). Protein signals were revealed with ECL Prime (Amersham, GE Healthcare, Boston, MA, USA) and detected by UVITEC (Eppendorf S.r.l., Hamburg, Germany). Each band’s intensity was evaluated using UVIDOC or the NIH Image J 1.8 software (National Institutes of Health, Bethesda, MD, USA). Optical density values of specific proteins were normalized to that of the loading control gene (βActin, GAPDH, or fibrillarin).

Patient’s enrollment and organotypic slice cultures (OSCs): PCa patients (*n* = 50) who underwent radical prostatectomy at the Department of Urology, Fondazione Policlinico Universitario A. Gemelli IRCCS-Università Cattolica, Rome Italy (from October 2015 to February 2020) were enrolled with the following inclusion criteria: (i) Clinically localized PCa at diagnosis and (ii) absence of androgen deprivation treatment/radiation therapy before surgery. This study was authorized by the ethical committee of IRCCS-Fondazione Policlinico Gemelli-Università Cattolica in Rome, Italy (protocol number: 25519/16; ID: 1247, date of approval July 7, 2016), and informed consent was obtained from each patient. All procedures were conducted in accordane with the principles expressed in the Declaration of Helsinki, the institutional regulation, and Italian laws and guidelines. OSCs were generated and RNA extracted and processed as described in [37]. Protein extraction was performed as described in [38].

PSA and citrate measurements in the extracellular medium. Determination of total (free and complexed) prostate-specific antigen (tPSA) was performed using the Elecsys Total PSA Kit (Roche Diagnostics S.p.A., Monza, Italy) in accordance with the manufacturer’s instructions.

Enzymatic colorimetric determination of citrate was performed using the Citric Acid in Urine Kit (CC00150, LTA s.r.l., Bussero (Milan), Italy) in accordance with the manufacturer’s instructions. Citrate concentration was measured 48 or 24 h after transfection for PCa cells and OSCs, respectively.

Computations: Two new ordinal variables were built: The “pathological stage” variable, assuming values from 1 to 5 (1 for pathological stage identified with “pT2a”, 2 for the pathological stage “pT2b”, 3 for “pT2c”, 4 for “pT3a”, 5 for “pT3b”. The pathological stage was in accordance with AJCC Cancer Staging Manual, 7th Edition, TNM 2010 [68]. The other variable was built to quantify the clinical tumor features (“adverse clinical features”) and was obtained as the sum of the parameters “lymphovascular invasion”, “residual surgical margins”, “extracapsular extension”, and “perineural invasion”, when resulted positive, with weights 1, 2, 3, and 0.5, respectively. The resulting variable “adverse clinical features” assumed values from 0 to 6.5.

Statistical analysis: Non-recurrence and recurrence patients were compared based on quantitative and qualitative variables using an independent *t*-test (accompanied by a Mann–Whitney U test) and a Fisher exact test.

To classify patients with recurrence and non-recurrence of disease, according to the values of the collected variables, a partial least-squares discriminant analysis (PLS-DA) was performed. A cross-validation approach was followed, and the optimal model with the optimal number of PLS components was derived. The Q2 value, the ROC AUC, and the derived misclassification error, by the classification Youden criterium, were used to quantify the classification goodness. A permutation approach, including 1000 random permutations, was used to associate a *p* level to the obtained Q2 value. The PLS-DA analysis was performed considering two models: The first model included either all the variables (or a combination of them) recorded in the clinical practice and the expression of some genes from the OSC analysis (new criterium); the second model included only those parameters measured in the standard clinical setting (classical criterium).

For all the other experiments, data were expressed as mean ± SEM or as fold change as indicated in figure legends. Significance was calculated using a non-parametric paired two-tailed Student’s *t*-test. Statistical analysis was performed using Sigma Plot 13.0 statistical software. *P*-values of < 0.05 were considered as significant.

## 5. Conclusions

Altogether, these data suggest that MALAT1 acts as a critical metabolic controller of prostate cancer. Indeed, MALAT1 targeting appears to restore sufficiently essential metabolic processes such as aerobic glycolysis in PCa cells and OSCs, thus resembling normal prostate epithelial cells. Although results obtained in the ex vivo model require validation in a larger cohort because of the limited number of samples evaluated, they indicate a relevant role played by MALAT1 in the metabolic reprogramming of prostate cancer cells. These results unravel the property of MALAT1 to regulate metabolic enzymes. Finally, MALAT1 and its metabolic targets showed the potential of predicting patient outcome when analyzed at the time of surgery.

Investigating the role of lncRNAs in cellular metabolism, particularly that of MALAT1, which exerts essential regulatory functions on the TCA cycle, may lead to the development of novel strategies to treat cancer. Specifically, developing tissue or tumor-specific knockdown of MALAT1 could be considered a valid strategy to restore metabolic control in transformed cells.

## Figures and Tables

**Table 1:** Clinical and histopathologic features of patients and their tumors.

Patient (OSC#)	Age	PSA (ng/mL)	Gleason Score	Pathologic Stage	Recurrence	Time of Recurrence (mo)
1 *	66	11.98	7 (4 + 3)	T2c Nx Mx	-	-
2	58	5	7 (3 + 4)	T2c Nx Mx	-	-
3 *	73	15.75	7 (3 + 4)	T3a N0 Mx	yes	7
4 *	56	2.15	7 (4 + 3)	T3a N0 Mx	yes	6
5 *	68	8.32	7 (4 + 3)	T2c Nx Mx	yes	8
6	73	12.8	7 (3 + 4)	T2c Nx Mx	-	-
7 *	69	4.72	7 (4 + 3)	T3b N0 Mx	yes	6
8	71	14	7 (3 + 4)	T2c Nx Mx	-	-
9	69	18.5	7 (3 + 4)	T3a N0 Mx	yes	17
10	75	10.25	7 (3 + 4)	T2c Nx Mx	-	-
11 *	65	11	7 (4 + 3)	T3b N0 Mx	yes	31
12 *	64	1.79	7 (3 + 4)	T2c Nx Mx	-	-
13	55	6.12	6 (3 + 3)	T2 Nx Mx	-	-
14	69	7.36	6 (3 + 3)	T2c Nx Mx	-	-
15	60	5.9	7 (4 + 3)	T2c N0 Mx	-	-
16 *	67	7.9	7 (3 + 4)	T2a Nx Mx	-	-
17	74	10.88	7 (4 + 3)	T2c N0 Mx	yes	7
18	74	6.2	7 (4 + 3)	T2c N0 Mx	-	-
19	63	4.7	6 (3 + 3)	T2c Nx Mx	-	-
20	75	4.88	7 (3 + 4)	T3a N0 Mx	-	-
21 *	59	6.8	6 (3 + 3)	T2c Nx Mx	-	-
22	60	4.9	6 (3 + 3)	T2c Nx Mx	-	-
23	75	6.3	7 (4 + 3)	T2c Nx Mx	yes	5
24	69	4.38	7 (3 + 4)	T2c N0 Mx	-	-
25	66	5.28	8 (4 + 4)	T2b Nx Mx	-	-
26	57	5.8	7 (3 + 4)	T2c Nx Mx	-	-
27 *	57	5.9	7 (3 + 4)	T2c Nx Mx	-	-
28 *	59	8.19	9 (4 + 5)	T2c N0 Mx	yes	9
29	72	15.04	7 (4 + 3)	T2 N0 Mx	-	-
30 *	75	11.48	7 (3 + 4)	T3a N0 Mx	yes	13
31	75	16.5	7 (3 + 4)	T2c N0 Mx	-	-
32	69	7.4	7 (4 + 3)	T3a N0 Mx	-	-
33	63	5.36	7 (3 + 4)	T2c Nx Mx	-	-
34 *	70	18.04	7 (3 + 4)	T2c N0 Mx	-	-
35	68	5.75	7 (4 + 3)	T3b N0 Mx	yes	6
36	63	14	7 (3 + 4)	T2c N0 Mx	-	-
37	61	8.5	7 (4 + 3)	T2c Nx Mx	-	5
38	57	18.69	7 (3 + 4)	T2c N0 Mx	-	-
39 *	55	6	7 (4 + 3)	T3b N0 Mx	yes	6
40	74	8.4	7 (3 + 4)	T2c Nx Mx	-	-
41	69	6	7 (3 + 4)	T2c Nx Mx	yes	7
42	78	15	7 (4 + 3)	T3a Nx Mx	-	-
43	67	5.5	7 (3 + 4)	T2c N0 Mx	-	-
44 *	67	13.5	7 (4 + 3)	T2c N0 Mx	-	-
45	69	11.3	9 (4 + 5)	T3b N0 Mx	yes	7
46 *	70	8.68	7 (3 + 4)	T2c N0 Mx	-	-
47	66	8.5	7 (4 + 3)	T3b N0 Mx	yes	5
48	61	18	7 (4 + 3)	T3a N0 Mx	-	-
49 *	65	6.6	7 (4 + 3)	T2c N0 Mx	-	-
50	69	11	7 (3 + 4)	T2c Nx Mx	-	-

* Significant depletion of MALAT1; *p* ≤ 0.05 MALAT1gapmer vs LacZgapmer (see Figure 7B).

## Supplementary Materials

The following are available online at https://www.mdpi.com/2072-6694/13/1/15/s1, Figure S1: Consequences of MALAT1 depletion in the PC3 cell line, Figure S2: MALAT1 level and depletion by gapmer in PCa cells (MALAT1/GAPDH), Figure S3: Effects of MALAT1 targeting on TCA cycle, Figure S4: Gene expression analysis at a different time point after MALAT1 silencing (DOWN), Figure S5: Gene expression analysis at a different time point after MALAT1 silencing (UP), Figure S6: EMT markers, Figure S7: C27IM cell line prostate-specific phenotype, Figure S8: Basal gene expression in PCa patient-derived OSCs, Figure S9: Uncropped gels for Figure 4A, Figure S10: Uncropped gels for Figure 4C, Figure S11: Uncropped gels for Figure 5A, Figure S12: Uncropped gels for Figure 5B, Figure S13: Uncropped gels for Figure 5C, Figure S14: Uncropped gels for Figure 7B, Figure S15: Uncropped gels for Figure 8F, Figure S16: Uncropped gels for Figure S3C, Figure S17: Uncropped gels for Figure S6, Figure S18: Uncropped gels for Figure S7A, Table S1: Concentration of oxidized and reduced nicotinamide adenine dinucleotide (NAD+, NADH), Table S2: Differential regulated genes gapmer MALAT1 vs. gapmer LacZ, Table S3: Concentration of adenosine diphosphate (ADP) and adenosine triphosphate (ATP), Table S4: Concentration of oxidized and reduced nicotinamide adenine dinucleotide phosphate (NADP+, NADPH), Table S5: Clinical, histopathological features and molecular biomarkers of prostate cancer patients and tumors in the whole sample: Non-recurrent vs. recurrent subgroups, Table S6: Clinical, histopathological characteristic and molecular biomarkers of patients and tumors in MALAT1-silenced OSCs subgroup.
