# Supplementary material for: Metabolic Reprogramming by Malat1 Depletion in Prostate Cancer
Source: Cancers (Basel). 2020 Dec 22;13(1):15. doi: 10.3390/cancers13010015 (PMC7801945; doi:10.3390/cancers13010015)
Supplement: Supplementary file 1 [file cancers-13-00015-s001.zip › cancers-1035998-Supplementary proof.pdf]

Supplementary Materials: Metabolic Reprogramming by Malat1 Depletion in Prostate Cancer (Stage 2)

Simona Nanni, Aurora Aiello, Chiara Salis, Agnese Re, Chiara Cencioni, Lorenza Bacci, Francesco Pierconti, Francesco Pinto, Cristian Ripoli, Paola Ostano, Silvia Baroni, Giacomo Lazzarino, Barbara Tavazzi, Dario Pugliese, PierFrancesco Bassi, Claudio Grassi, Simona Panunzi, Giovanna Chiorino, Alfredo Pontecorvi, Carlo Gaetano, Antonella Farsetti

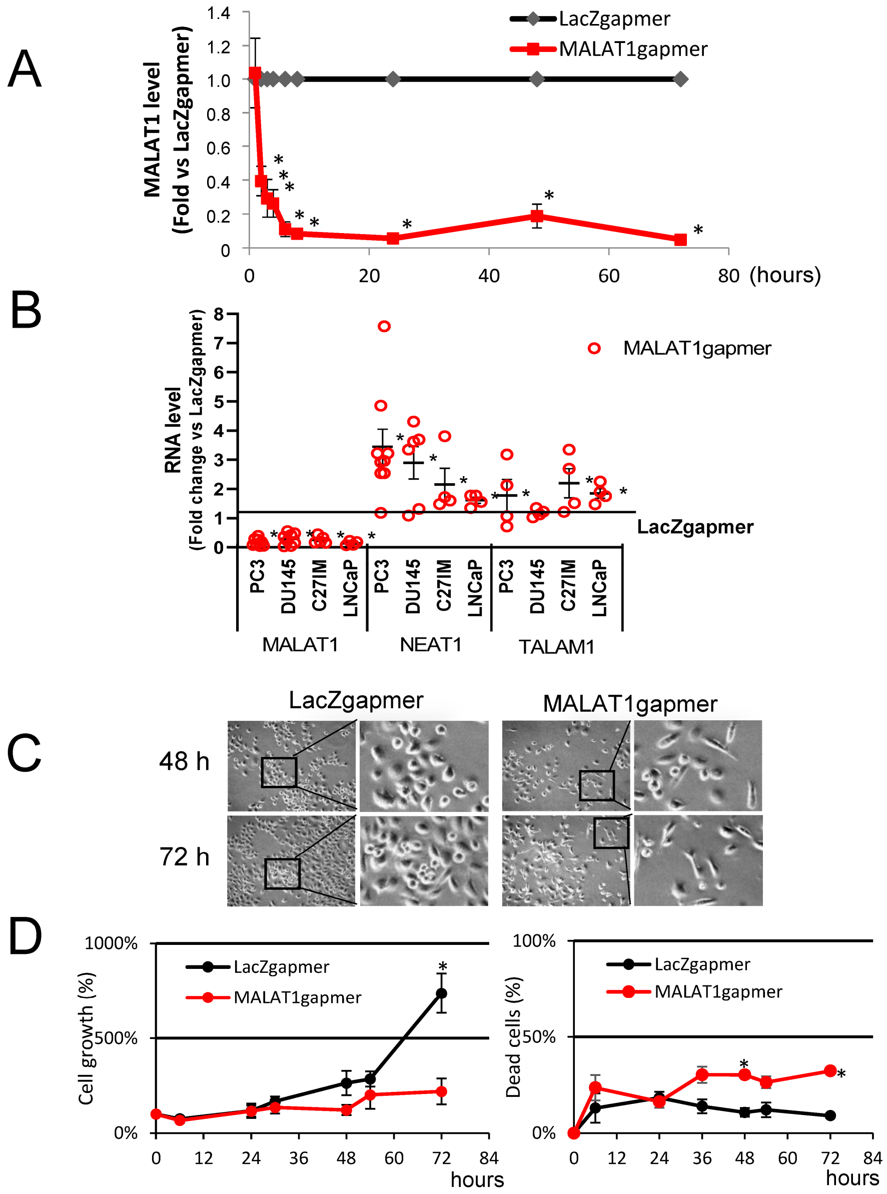

Figure S1. Consequences of MALAT1 depletion in PC3 cell line. (**A**) MALAT1 expression levels quantified by qRT-PCR in PC3 cells transfected with specific (MALAT1) or control (LacZ) gapmers at different time points after transfection (1–72h). Results are plotted as fold induction vs LacZgapmer (placed to 1 and depicted as black line). Individual values (*n* = 3) with mean ± SEM are showed. (**B**) LncRNAs expression levels (MALAT1, NEAT1 and TALAM1) were quantified by qRT-PCR in prostate cancer cell lines (PC3, DU145, C27IM and LNCaP) at 72 h after transfection with MALAT1 or LacZ gapmers. Results are plotted as fold induction vs LacZgapmer (placed to 1 and depicted as black line). Individual values (*n* = 4 to 8) with mean ± SEM are showed. (**C**) Representative images in

white field under magnification 20x of PC3 cells at 48h or 72h after transfection with MALAT1gapmer or LacZgapmer. Black rectangle indicated zoom (3x) of selected areas in right panels. (**D**) Viability of PC3 cells transfected with (MALAT1gapmer) or LacZgapmer was determined by trypan blue dye exclusion assay at different time points after transfection (1–72h). Cell growth and cell death, expressed as percentage, represent mean ± SEM (n = 3). Statistical significance was determined by non-parametric paired two-tailed Student's t-test. *$p \leq 0.05$ MALAT1gapmer vs LacZgapmer.

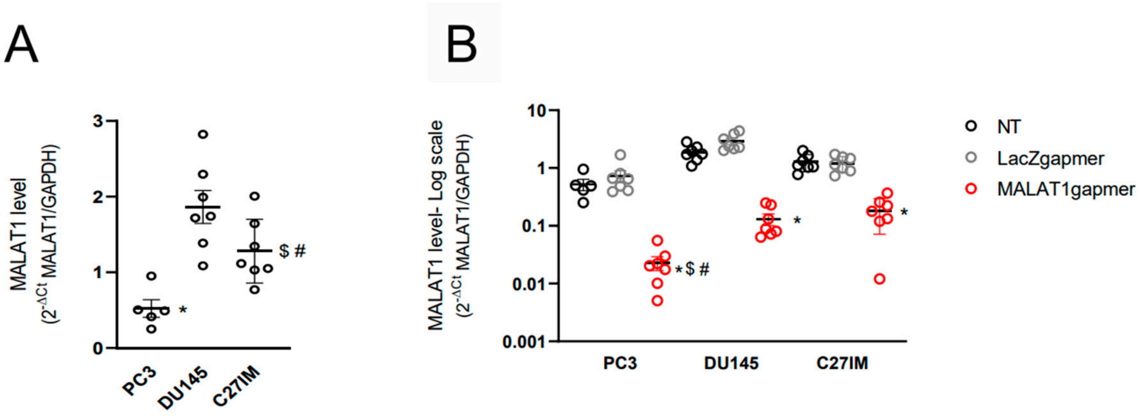

Figure S2. MALAT1 level and depletion by gapmer in PCa cells (MALAT1/GAPDH). (**A**) MALAT1 basal level levels quantified by qRT-PCR in PC3, DU145 and C27IM cells. Individual values with mean +/- SEM are showed. Statistical significance was determined by non-parametric paired two-tailed Student's t-test. *$p \leq 0.05$ PC3 vs DU145. $^{\$}p \leq 0.05$ C27IM vs DU145. $^{\#}p \leq 0.05$ PC3 vs C27IM. (**B**) MALAT1 expression in PCa cells (B) transfected with specific (MALAT1), control (LacZ) gapmers or no ga. Results are plotted as fold induction vs LacZgapmer (placed to 1 and depicted as black line). Individual values (n = 5–8) with mean ± SEM are showed. Statistical significance was determined by nonparametric paired two-tailed Student's t-test. *$p \leq 0.05$ MALAT1gapmer vs LacZgapmer. $^{\$}p \leq 0.05$ PC3 vs DU145. $^{\#}p \leq 0.05$ PC3 vs C27IM.

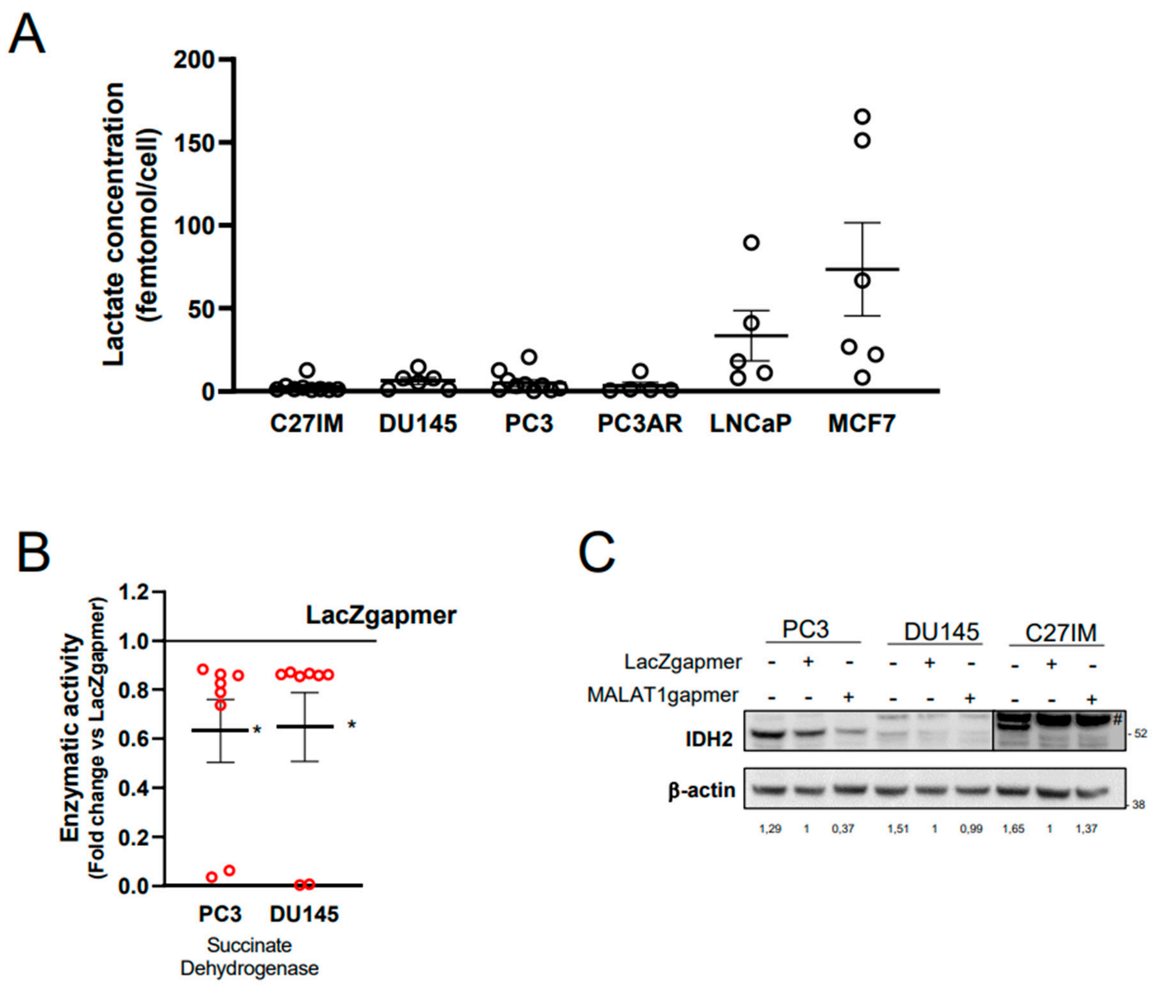

Figure S3. Effects of MALAT1 targeting on TCA cycle. (**A**) Lactate concentration analysis by colorimetric assay in cell culture supernatant of PC3, PC3AR, DU145, LNCaP, C27IM and MCF7 cells (*n* = 5 to 10). (**B**) Enzymatic activity of Succinate dehydrogenase was measured in PC3 and DU145 cells 24h after transfection with specific (MALAT1) or control (LacZ) gapmer. Results are plotted as fold induction vs LacZgapmer (placed to 1 and depicted as black line). Individual values (*n* = 8) with mean ± SEM are showed. (**C**) Representative western blot and densitomentric analysis for IDH2 assessed in PC3, DU145 and C27IM cells untransfected or transfected with specific gapmers for MALAT1 or LacZ as control. b-actin was used as loading control. Numbers represent Fold Induction vs LacZgapmer of densitometric bands normalized to loading control. Molecular weight marker is indicated. # Higher exposure. Statistical significance was determined by non-parametric paired two-tailed Student's t-test. *$p \leq 0.05$ MALAT1gapmer vs LacZgapmer.

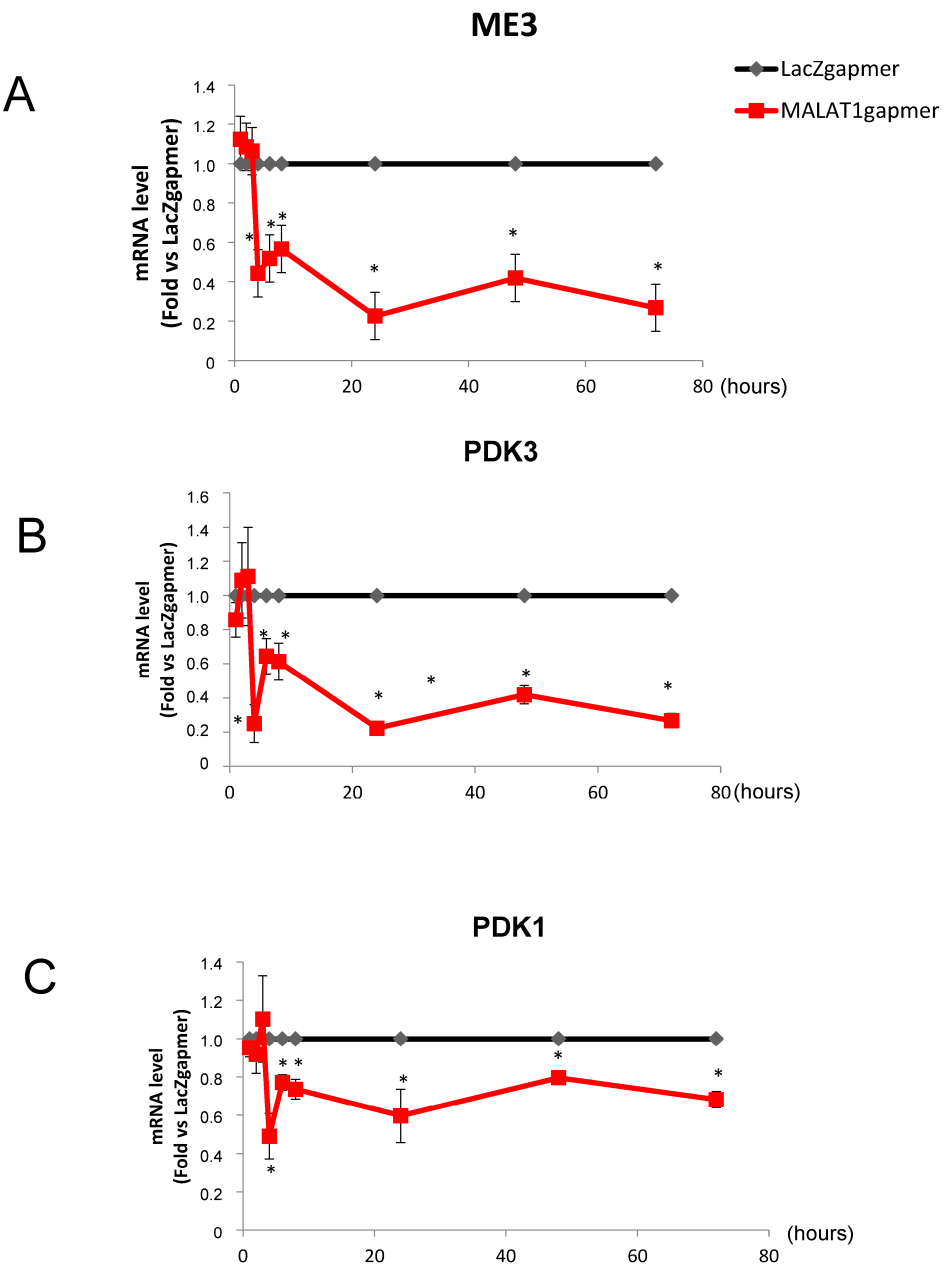

Figure S4. Gene expression analysis at different time point after MALAT1 silencing (DOWN). (**A**–**C**) ME3, PDK3 and PDK1 expression levels quantified by qRT-PCR in PC3 cells transfected with specific (MALAT1) or control (LacZ) gapmer at different time points after transfection (1–72h). Results are plotted as fold induction vs LacZgapmer (placed to 1 and depicted as black line). Individual values ($n = 3$) with mean ± SEM are showed. Statistical significance was determined by non-parametric paired two-tailed Student's t-test. *$p \leq 0.05$ MALAT1gapmer vs LacZgapmer.

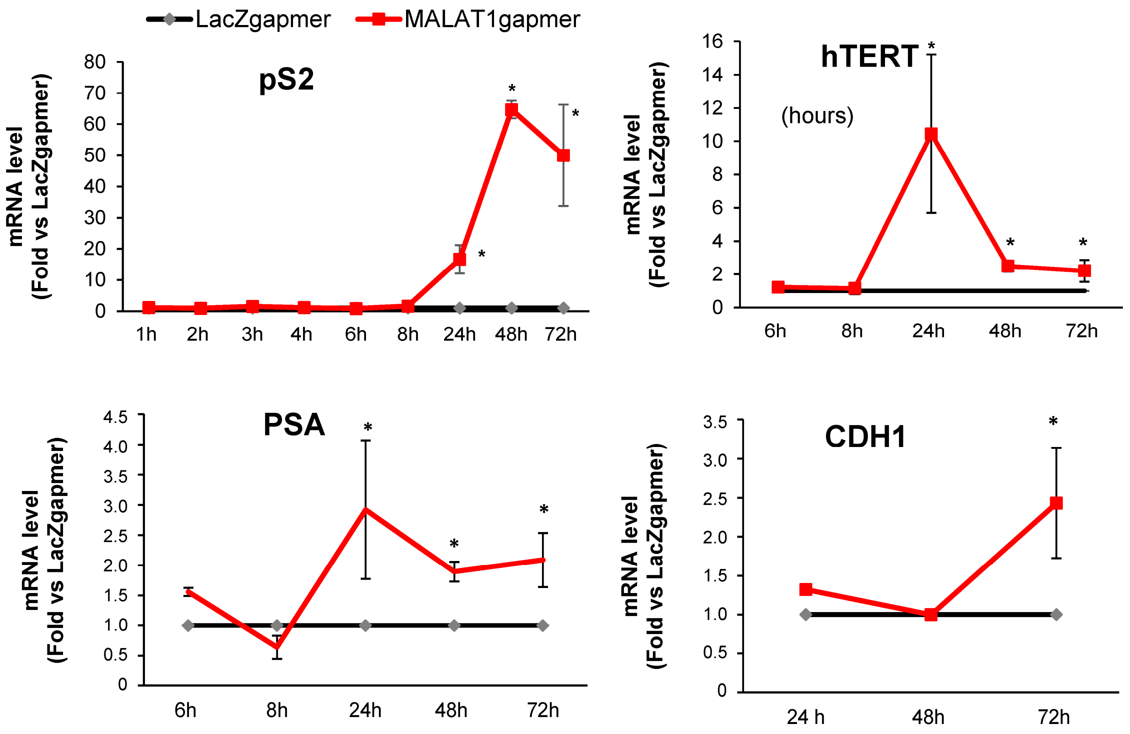

Figure S5. Gene expression analysis at different time point after MALAT1 silencing (UP). pS2, hTERT, PSA and CDH1 expression levels quantified by qRT-PCR in PC3 cells transfected with specific (MALAT1) or control (LacZ) gapmer at different time points after transfection (1–72h). Results are plotted as fold induction vs LacZgapmer (placed to 1 and depicted as black line). Individual values ($n = 3$) with mean ± SEM are showed. Statistical significance was determined by non-parametric paired two-tailed Student's t-test. *$p \leq 0.05$ MALAT1gapmer vs LacZgapmer.

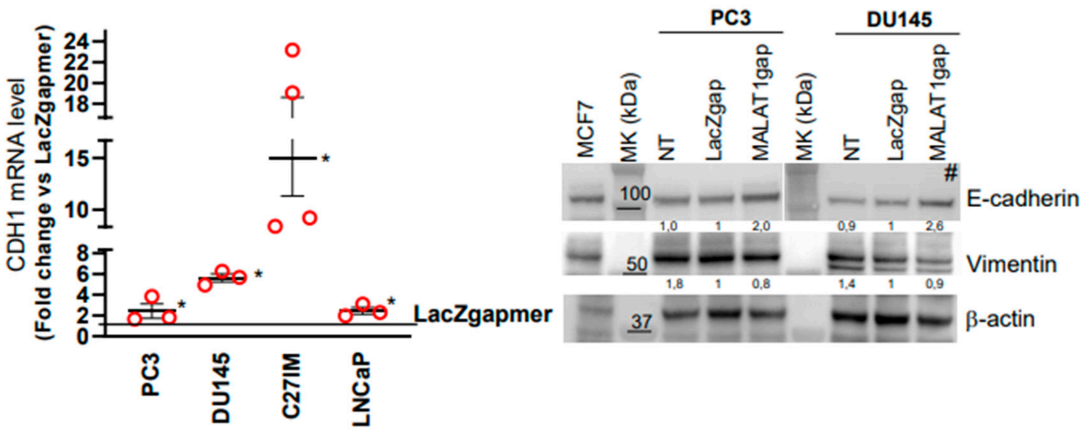

Figure S6. EMT Markers. Left panel: CDH1 expression levels quantified by qRT-PCR in PC3, DU145, C27IM and LNCaP cells transfected with specific (MALAT1) or control (LacZ) gapmer at 72h after transfection. Results are plotted as fold induction vs LacZgapmer (placed to 1 and depicted as black line). Individual values ($n = 3$) with mean ± SEM are showed. Statistical significance was determined by non-parametric paired twotailed Student's t-test. *$p \leq 0.05$ MALAT1gapmer vs LacZgapmer. Right panel: Analysis by western blot of Ecadherin and vimentin. b-actin served as loading control in PC3 and DU145 cells untransfected or transfected with gapmer to MALAT1 or LacZ. White line indicates non-contiguous lanes run on same gel. Numbers represent Fold Induction vs LacZgapmer of

densitometric bands normalized to loading control. # higher exposure. Molecular weight marker is indicated.

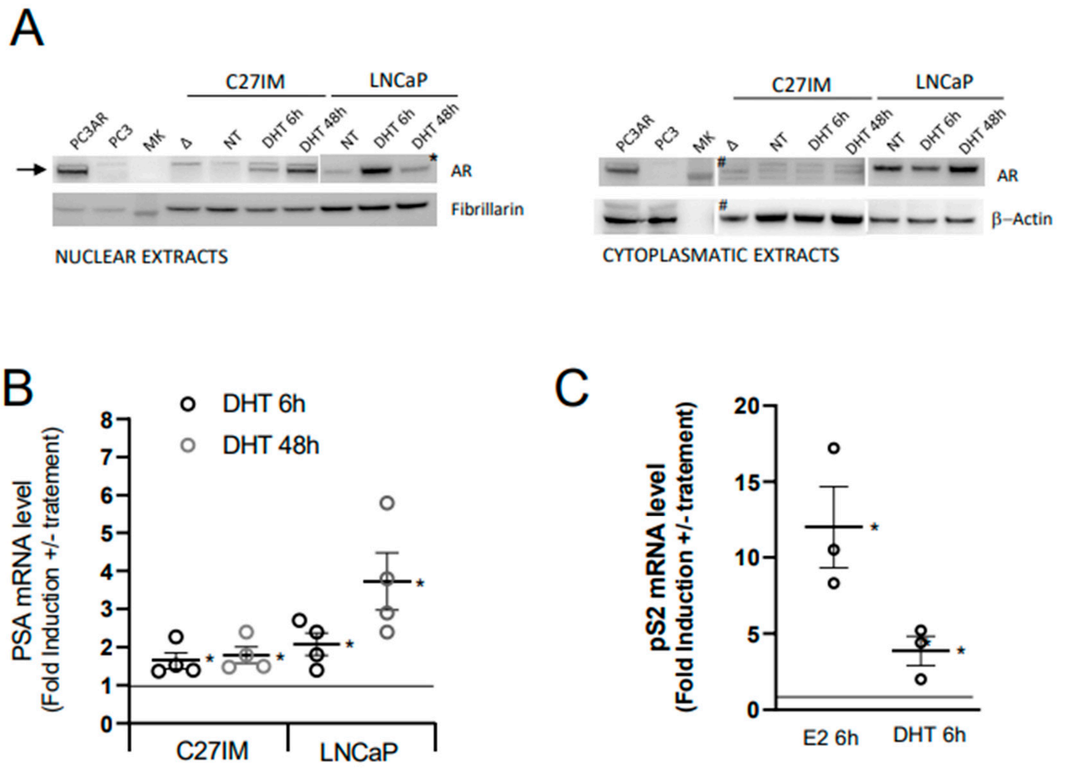

Figure S7. C27IM cell line prostate-specific phenotype. (**A**) Androgen receptor (AR) analysis by western blot using fractionated cells extracts (nuclear or cytoplasmatic extracts, respectively) of C27IM and LNCaP cells cultured in the absence (NT) or presence of DHT for 6 or 48h. C27IM cells cultured in non-deprived serum are shown (**B**). PC3 and AR-transfected PC3 (PC3-AR) cells were used as negative and positive control, respectively. Fibrillarin and b-Actin were used as loading control. White lines indicate non-contiguous lanes run on the same gel. * Lower exposure. # Higher exposure. Molecular weight marker is indicated. (**B**) PSA mRNA level was quantified in C27IM (left) and LNCaP (right) cells treated with DHT for the time indicated or vehicle as control. (**C**) pS2 mRNA level was quantified in C27IM cells treated with 17b-estradiol (E2) or DHT or veichle as control. Results are plotted as fold induction ± treatment (control was placed to 1 and depicted as black line). Individual values with mean ± SEM are showed. Statistical significance was determined by non-parametric paired two-tailed Student's t-test. **p* ≤ 0.05 treatment vs control.

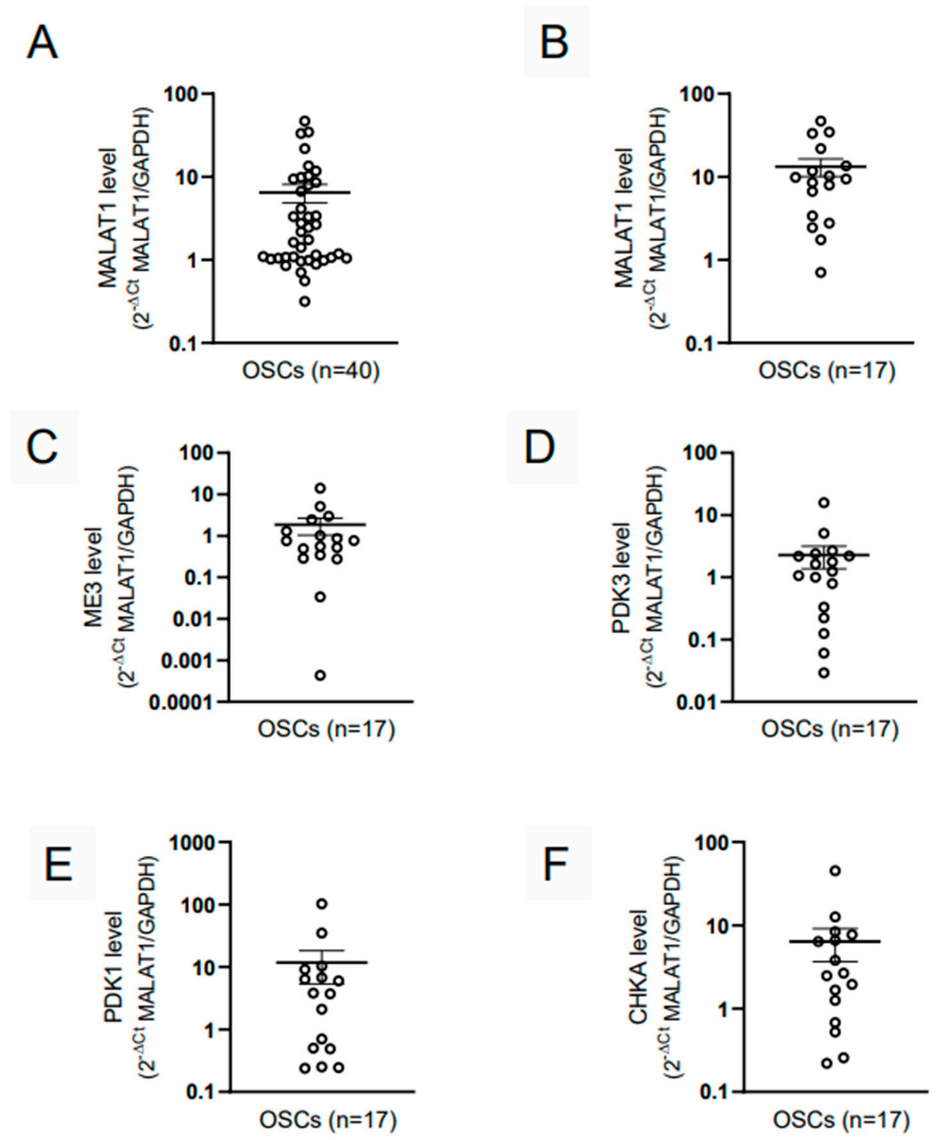

Figure S8. Basal gene expression in PCa-patient derived OSCs. (**A**) MALAT1 basal level levels quantified by qRT-PCR in whole cohort of OSCs (n = 40). (**B**–**F**) MALAT1, ME3, PDK3, PDK1 and CHKA expression in OSC with a significant MALAT1 depletion (n = 17). Individual values with mean ± SEM are showed.

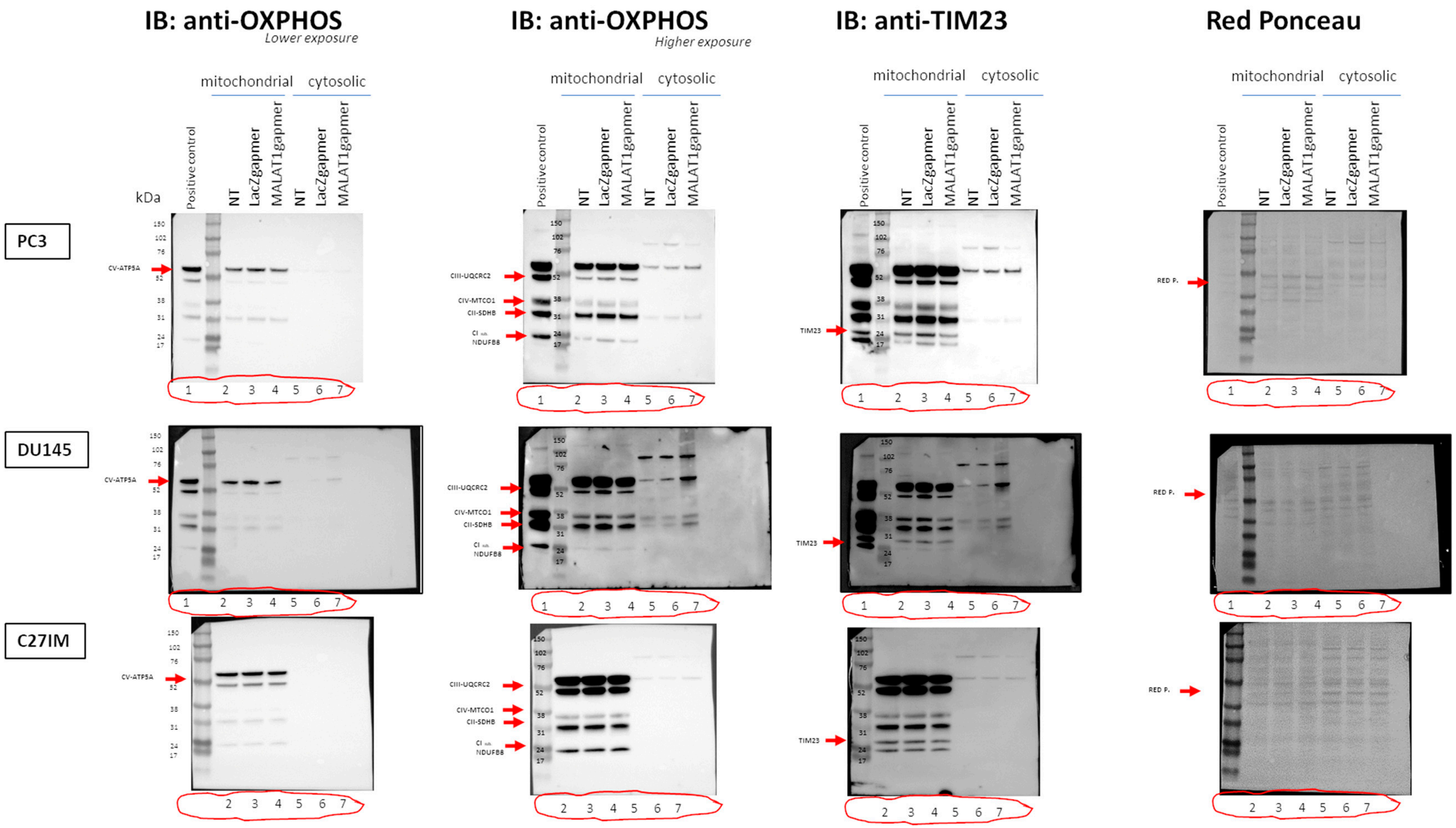

Figure S9. Uncropped gels for Figure 4A.

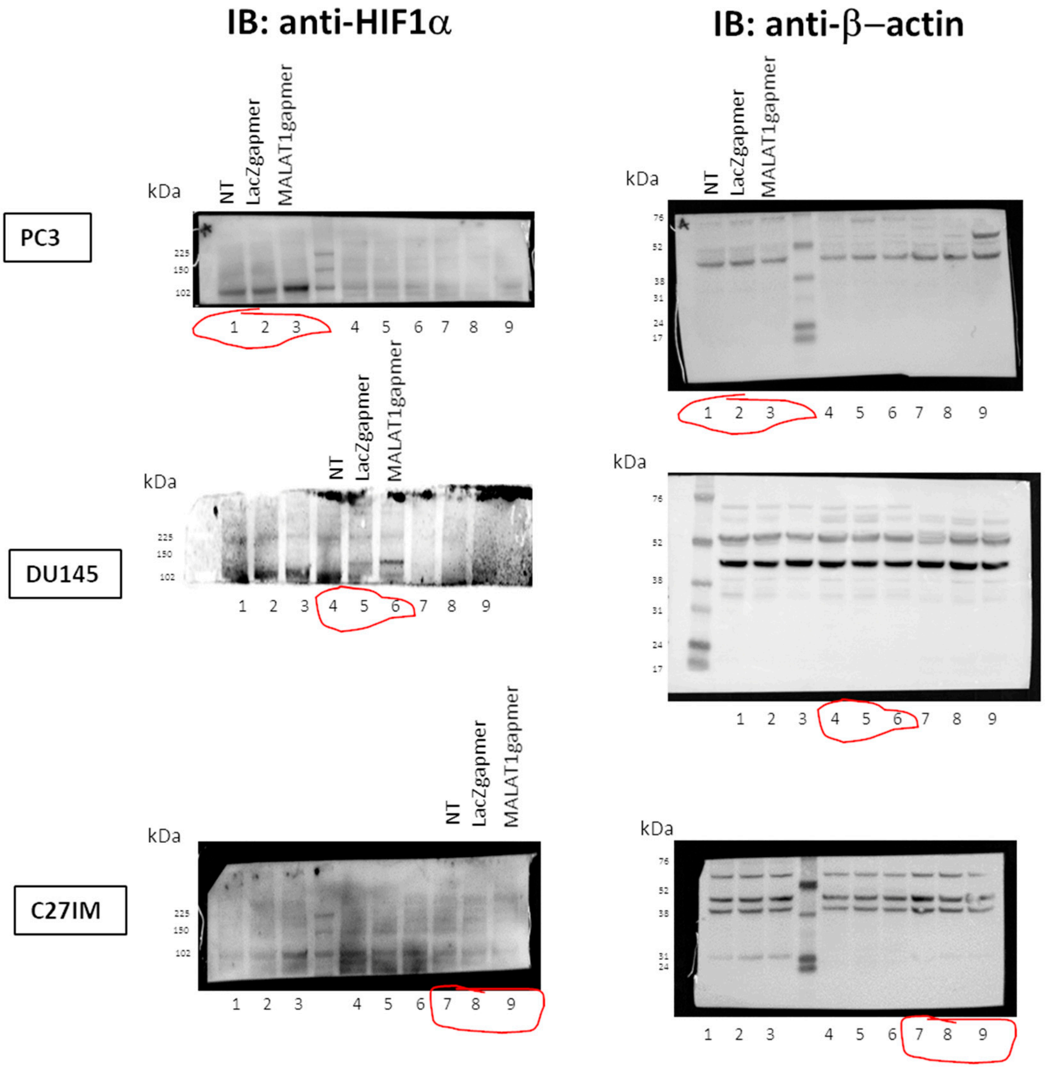

Figure S10. Uncropped gels for Figure 4C.

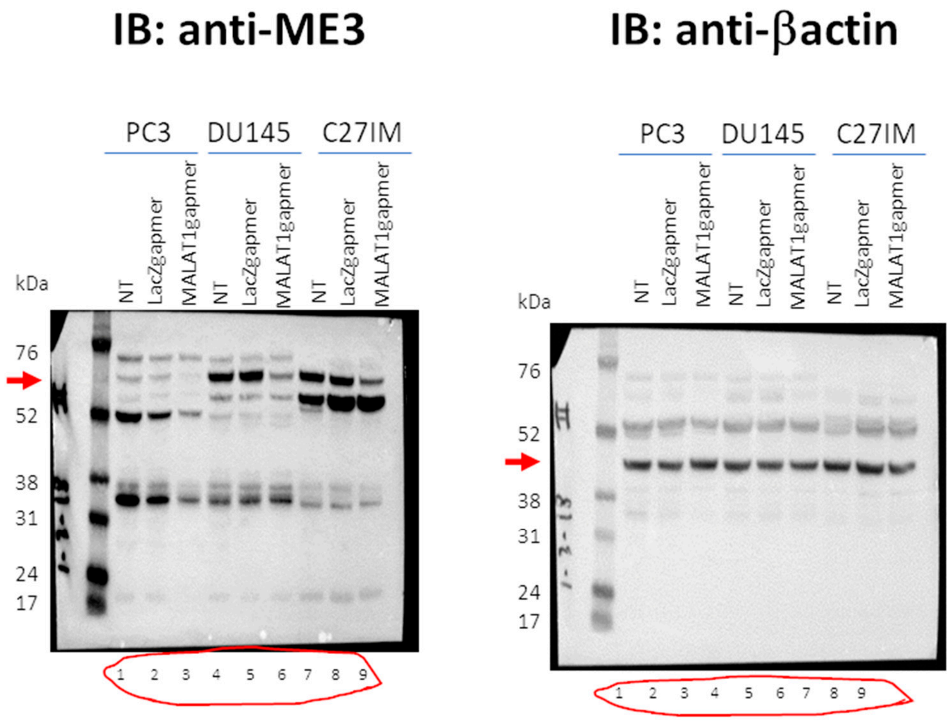

Figure S11. Uncropped gels for Figure 5A.

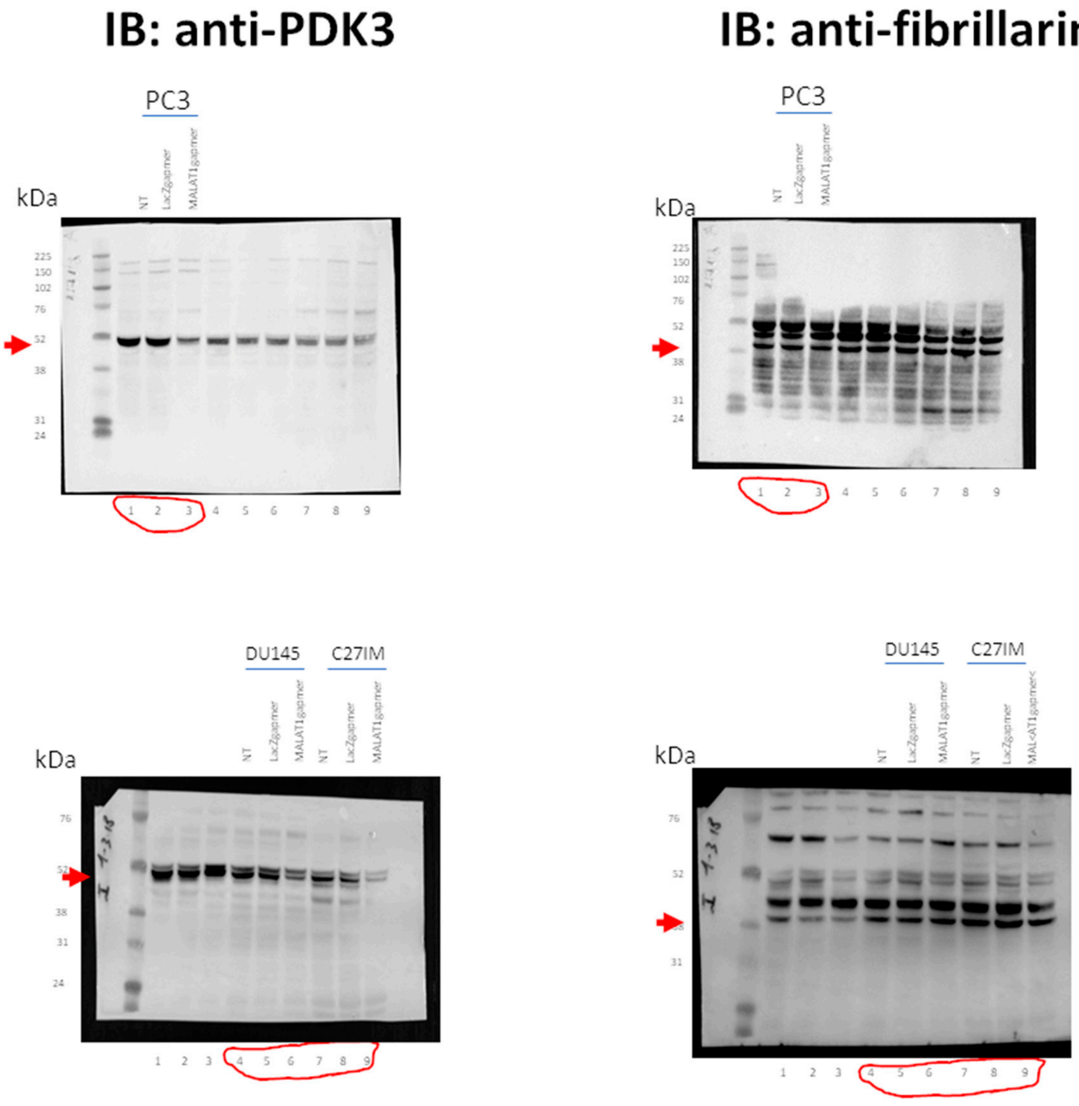

Figure S12. Uncropped gels for Figure 5B.

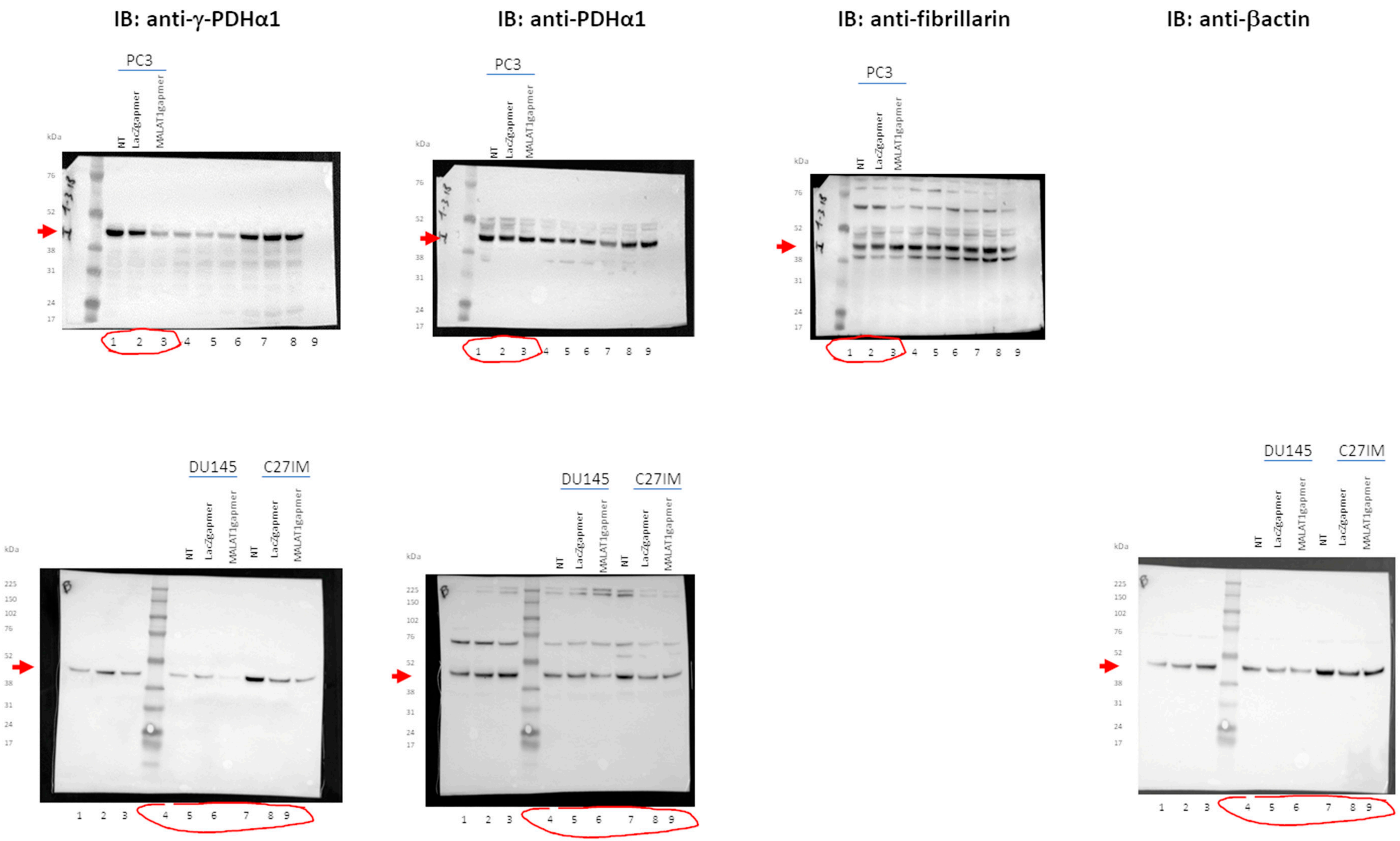

Figure S13. Uncropped gels for Figure 5C.

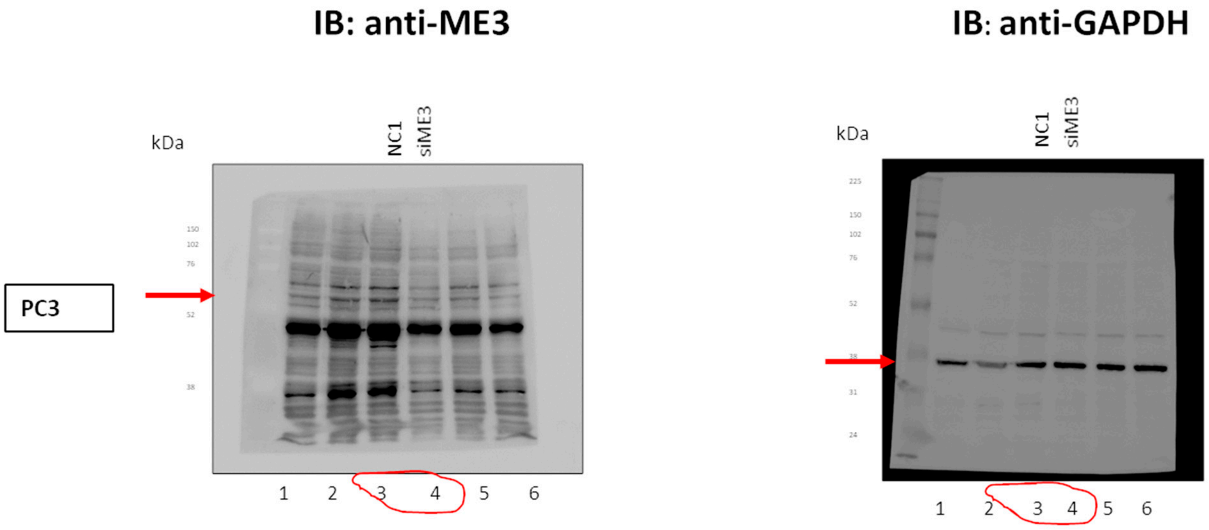

Figure S14. Uncropped gels for Figure 7B.

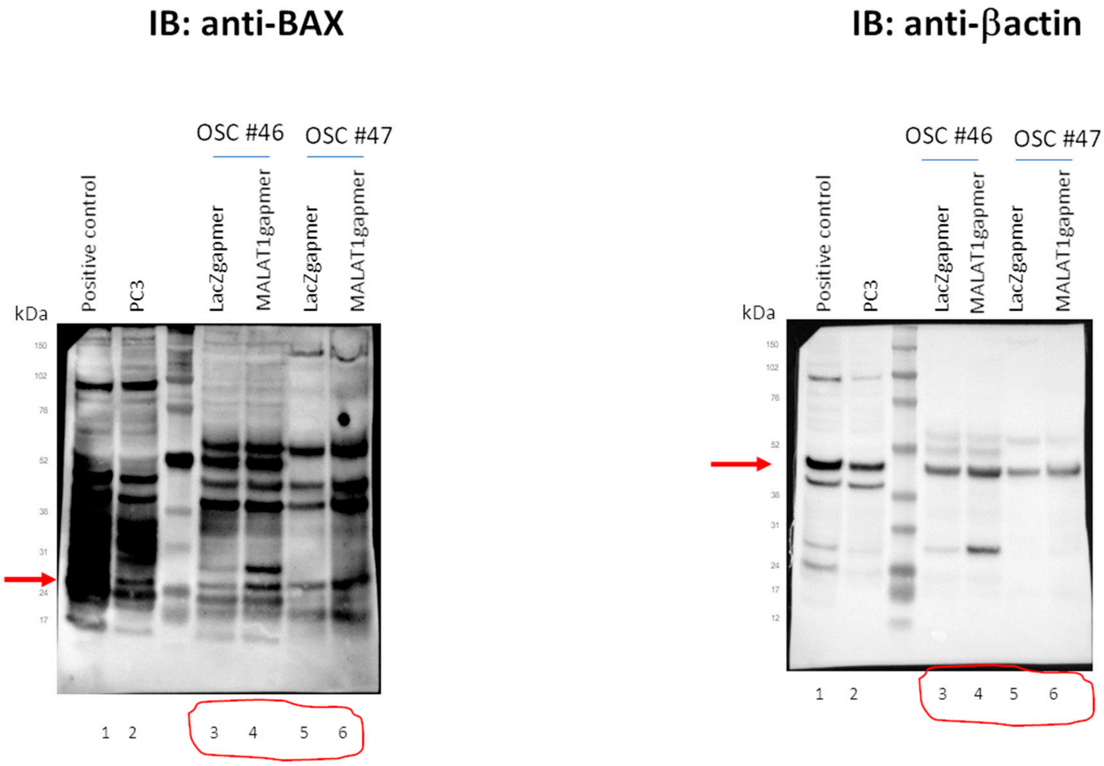

Figure S15. Uncropped gels for Figure 8F.

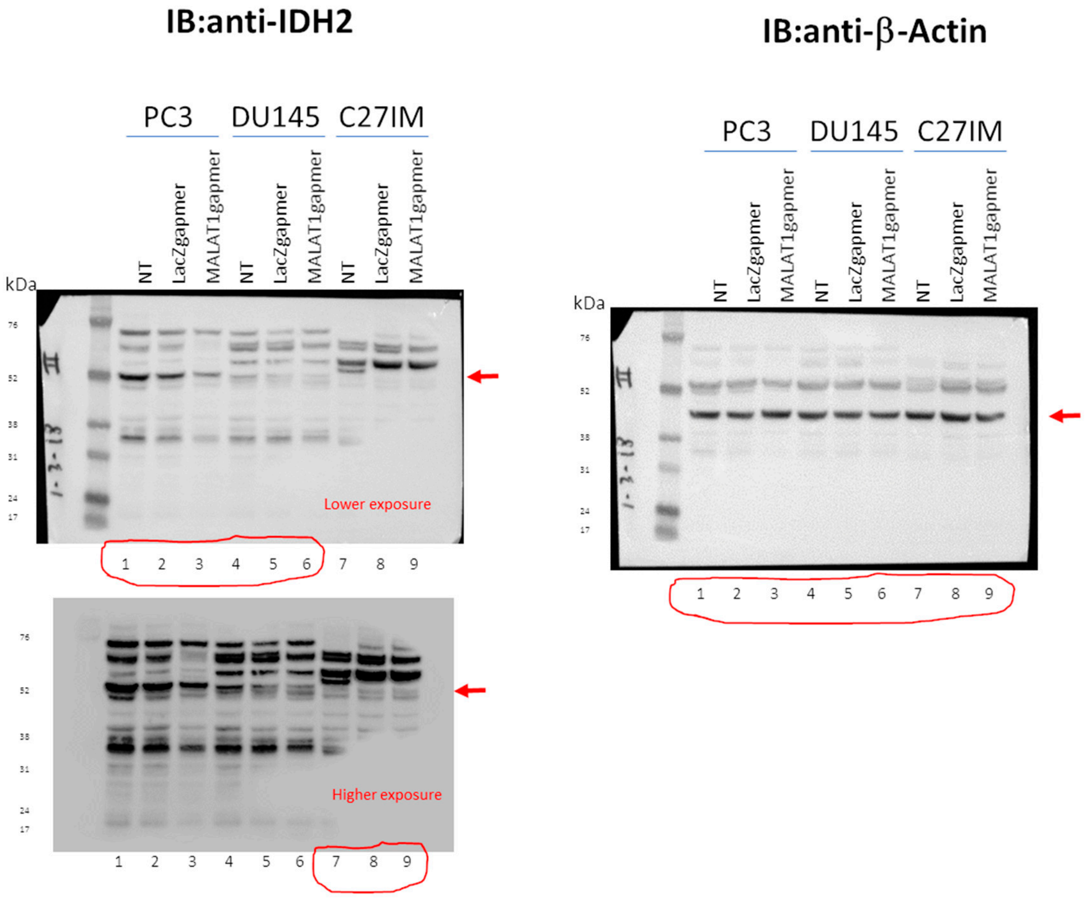

Figure S16. Uncropped gels for Figure S3C.

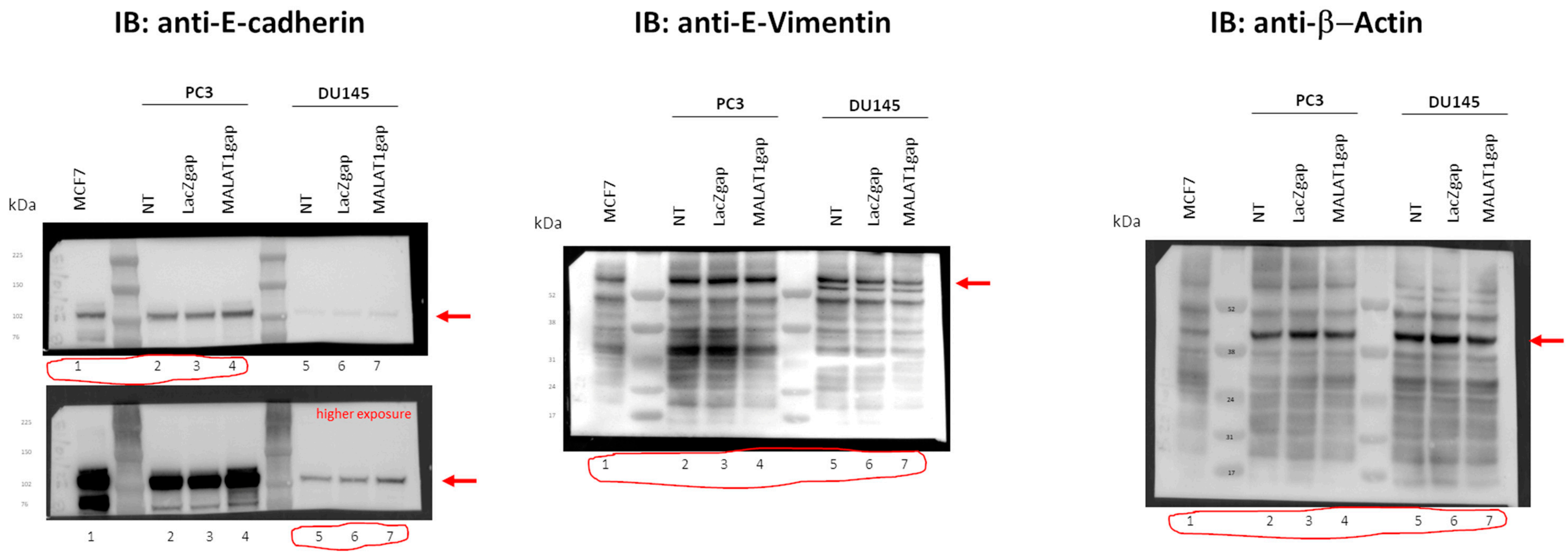

Figure S17. Uncropped gels for Figure S6.

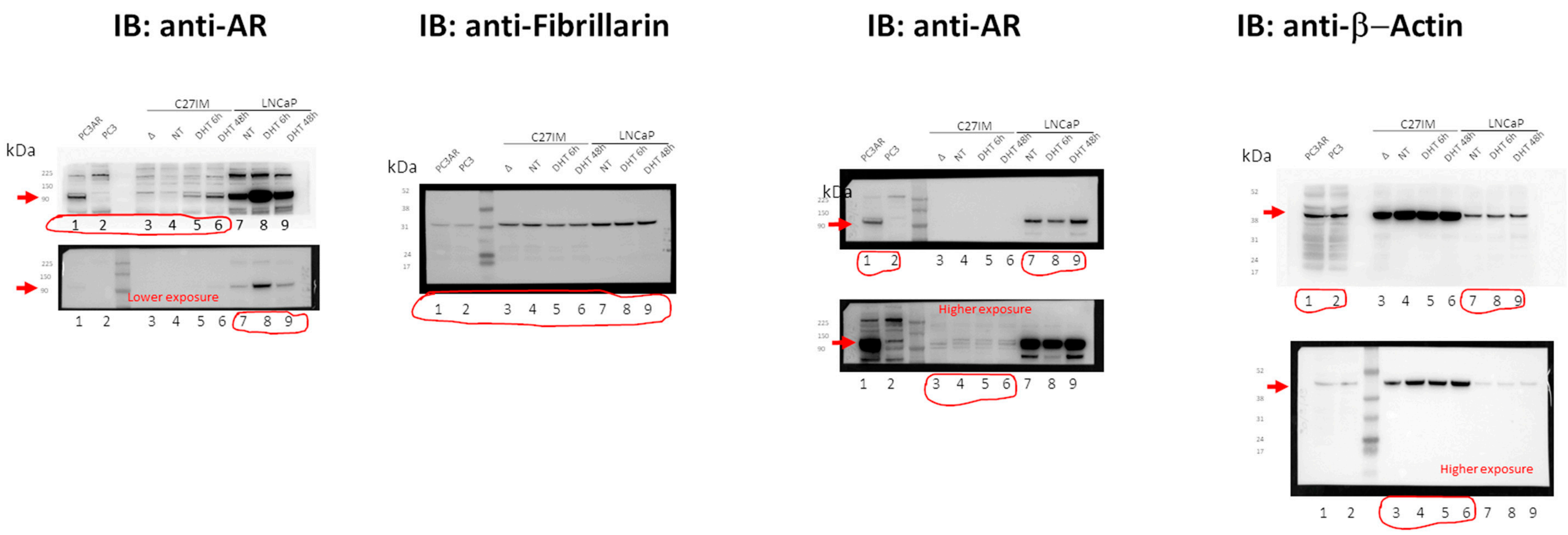

Figure S18. Uncropped gels for Supplemental Figure 7A.

Table S1. Concentration of oxidized and reduced Nicotinamide Adenine Dinucleotide (NAD^+, NADH) determined by HPLC in PC3 cell extracts transfected with specific (MALAT1) or control (LacZ) gapmer at 24h after transfection ($n = 4$, mean ± S.D). Statistical significance was determined by two-tailed Student's t-test. *$p \leq 0.001$ MALAT1gapmer vs LacZgapmer.

	LacZgapmer	MALAT1gapmer
**NAD^+** (nmol/10^6 cells)	3.240 ± 0.257	3.745 ± 0.337
**NADH (**nmol/10^6 cells)	0.186 ± 0.022	0.303 ± 0.021*
**NAD^+/NADH**	17.530 ± 1.789	12.335 ± 0.312*

Table S2 (available in separate Excel sheet): Table S2: Differential regulated genes gapmer MALAT1 vs. gapmer LacZ

Table S3. Concentration of Adenosine Diphosphate (ADP) and Adenosine Triphosphate (ATP) determined by HPLC in PC3 cell extracts transfected with specific (MALAT1) or control (LacZ) gapmers at 24h after transfection ($n = 4$, mean ± S.D).

	LacZgapmer	MALAT1gapmer
ATP (nmol/10^6 cells)	12.148 ± 2.053	12.174 ± 1.248
ADP (nmol/10^6 cells)	1.846 ± 0.438	1.720 ± 0.197
ATP/ADP	6.697 ± 0.721	7.145 ± 1.073

Table S4. Concentration of Oxidized and reduced Nicotinamide Adenine Dinucleotide Phosphate (NADP+, NADPH) determined by HPLC in PC3 cell extracts transfected with specific (MALAT1) or control (LacZ) gapmer at 24h after transfection ($n = 4$, mean ± S.D). Statistical significance was determined by two-tailed Student's t-test. *$p \leq 0.001$ MALAT1gapmer vs LacZgapmer.

	LacZgapmer	MALAT1gapmer
$NADP^+$ (nmol/10^6 cells)	0.343 ± 0.048	0.404 ± 0.117
NADPH (nmol/10^6 cells)	0.234 ± 0.024	0.124 ± 0.036*
$NADP^+$/NADPH	1.485 ± 0.287	3.258 ± 0.316*

Table S5. Clinical, histopatological features and molecular biomarkers of prostate cancer patients and tumors in the whole sample: Non-Recurrent vs Recurrent subgroups.

	**Non Recurrent ($n = 28$)**		**Recurrent ($n = 12$)**		**Total ($n = 40$)**		
	**Mean**	**SD**	**Mean**	**SD**	**Mean**	**SD**	***p***
Age (year)	65.39	5.88	67.17	7.11	65.93	6.24	0.457
(median, range)	65.50	20.00	68.50	20.00	66.50	20.00	0.383
SerumPSA (ng/mL)	9.39	4.81	8.05	3.70	8.99	4.50	0.345
(median, range)	7.40	16.90	7.25	13.60	7.40	16.90	0.575
Pathological Gleason Score	6.89	0.42	7.17	0.58	6.98	0.48	0.157
(median, range)	7.00	2.00	7.00	2.00	7.00	3.00	0.154
ISUP score	2.25	0.80	3.3	0.79	2.5	0.88	0.006
(median, range)	2	3	3	3	2	4	0.005
Pathological.stage (TNM 2010)	2.86	0.71	3.92	0.90	3.18	0.90	0.002
(median, range)	3.00	3.00	4.00	2.00	3.0	4.00	0.000
Adverse.clinical.features	1.00	1.18	2.83	1.67	1.55	1.57	0.003
(median, range)	0.50	3.50	3.00	5.00	0.50	5.50	0.001
MALAT1*	8.10	9.32	10.58	8.97	8.84	9.17	0.438
(median, range)	5.85	46.70	8.46	31.16	6.31	46.70	0.273
PSA*	6.51	14.49	19.05	45.41	10.28	27.58	0.368
(median, range)	1.76	70.74	6.59	161.88	2.30	161.94	0.213
PS2*	43.52	120.38	8.07	11.58	32.88	101.69	0.134
(median, range)	3.33	480.44	2.88	36.51	2.92	480.44	0.631
ME3*	0.81	1.05	1.97	3.84	1.16	2.28	0.323
(median, range)	0.46	5.08	0.71	13.68	0.59	13.96	0.115

PDK3*	1.56	1.62	2.75	4.17	1.92	2.65	0.358
(median, range)	1.35	5.71	1.45	15.24	1.35	15.79	0.273
CHKA*	4.36	3.71	8.00	12.10	5.48	7.40	0.328
(median, range)	4.22	12.61	4.39	44.62	4.26	45.29	0.480
PDK1*	9.03	19.56	7.35	9.22	8.51	16.94	0.716
(median, range)	4.92	102.80	6.10	34.36	5.30	102.80	0.499
Lymphovascular invasion	*N*	%	*N*	%	*N*	%	$^{\$}p$ value
Negative	27	73.0	10	27.0	37	92.5	0.21
Positive	1	33.3	2	66.7	3	7.5	
Residual surgical margins							
pR0	23	74.2	8	25.8	31	77.5	0.41
pR1	5	55.6	4	4.44	9	22.5	
Extracapsular extension							
Negative	26	81.3	6	18.1	32	80	0.005
Positive	2	25.0	6	75.0	8	20	
Perineural invasion							
Negative	6	100.0	0	0.0	6	14.0	0.15
Positive	22	59.5	15	40.5	37	86.0	
Seminal vescicles invasion							
Negative	28	77.8	8	22.2	36	90	0.005
Positive	0	0.0	4	100.0	4	10	

$^{\$}p$ value from Fisher exact test; * Gene basal level 2^{-DCt} MALAT1/GAPDH.

Table S6. Clinical, histopatological characteristic and molecular biomarkers of patients and tumors in MALAT1-silenced OSCs subgroup.

	Non Recurrent (*n* = 9)		Recurrent (*n* = 8)		Total (*n* = 17)		
	**Mean**	**SD**	**Mean**	**SD**	**Mean**	**SD**	***p***
Age (year)	65.00	4.47	65.00	7.62	65.00	5.95	1.000
(median, range)	66.00	13.00	66.50	20.00	66.00	20.00	0.962
Serum PSA (ng/mL)	9.02	4.80	8.45	4.29	8.75	4.43	0.799
(median, range)	7.90	16.25	8.26	13.60	8.19	16.25	0.888
Pathological Gleason Score	6.89	0.33	7.25	0.71	7.06	0.56	0.217
(median, range)	7.00	1.00	7.00	2.00	7.00	3.00	0.198
ISUP score	2.22	0.67	3.00	0.93	2.59	0.87	0.072
(median, range)	2	2	3	3	3	4	0.072
Pathological.stage (TNM 2010)	2.78	0.67	4.13	0.83	3.41	1.00	0.003
(median, range)	3.00	2.00	4.00	2.00	3.00	4.00	0.003
Adverse.clinical.features	0.94	1.12	3.50	1.51	2.15	1.86	0.002
(median, range)	0.50	3.50	3.50	5.00	2.50	5.50	0.005
MALAT1*	10.80	14.32	13.06	10.18	11.86	12.21	0.711
(median, range)	7.96	46.20	9.62	31.16	9.39	46.20	0.370
MALAT1 ratio$^{\$}$	0.52	0.27	0.61	0.21	0.57	0.24	0.445
(median, range)	0.64	0.80	0.73	0.49	0.70	0.82	0.311
PSA*	2.56	2.29	4.85	5.14	3.63	3.94	0.273
(median, range)	2.71	6.37	4.44	14.85	3.00	14.90	0.370
PSA ratio$^{\$}$	1.24	1.30	3.11	3.21	2.12	2.50	0.158
(median, range)	0.73	3.75	2.69	10.13	1.73	10.23	0.228
PS2*	53.22	147.56	5.45	8.67	30.74	107.35	0.361
(median, range)	2.05	446.33	1.85	25.52	2.05	446.59	0.423
PS2 ratio$^{\$}$	2.71	4.23	4.53	5.48	3.57	4.79	0.463
(median, range)	0.70	11.55	2.43	15.62	0.95	15.62	0.606
ME3*	1.22	1.64	2.58	4.67	1.86	3.37	0455
(median, range)	0.49	5.08	0.77	13.68	0.76	13.96	0.481
ME3 ratio$^{\$}$	1.37	1.95	2.01	3.11	1.67	2.50	0.625

(median, range)	0.72	6.28	0.87	9.09	0.72	9.42	0.606
PDK3*	1.34	1.71	3.32	5.08	2.27	3.71	0.325
(median, range)	0.33	5.12	1.51	15.00	1.25	15.79	0.236
PDK3 ratio$	1.21	0.94	1.90	2.37	1.54	1.74	0.465
(median, range)	0.98	2.68	1.08	7.09	0.98	7.43	0.606
CHKA*	3.42	4.43	8.89	14.87	6.16	1097	0.348
(median, range)	1.18	12.40	4.12	44.62	2.57	45.08	0.234
CHKA ratio$	1.22	1.18	0.90	0.37	1.06	0.86	0.489
(median, range)	0.77	3.48	0.84	1.08	0.83	3.48	0.798
PDK1*	15.36	35.48	8.17	11.29	11.76	25.71	0.599
(median, range)	2.12	102.56	5.09	34.36	3.78	102.56	0.328
PDK1 ratio$	0.71	0.39	1.22	0.94	0.97	0.74	0.185
(median, range)	0.65	1.28	0.87	2.84	0.68	3.01	0.279

* Gene basal level 2^{-DCt} MALAT1/GAPDH; $ fold induction MALAT1gapmer vs LacZgapmer.
